# Urinary Biomarkers Of Kidney Function As Predictors Of Cardiovascular Health: A Systematic Review

**DOI:** 10.1007/s11906-025-01328-5

**Published:** 2025-02-21

**Authors:** A. Degenaar, R. Kruger, A. Jacobs, C. M. C. Mels

**Affiliations:** 1 Hypertension in Africa Research Team (HART), Private Bag X1290, North-West University, Potchefstroom, 2520 South Africa; 2 MRC Research Unit: Hypertension and Cardiovascular Disease, North-West University, Potchefstroom, South Africa

**Keywords:** Alpha-1 microglobulin (uA1M), Cardiovascular health, CKD273 classifier, Hypertension, Kidney function, Neutrophil gelatinase-associated lipocalin (uNGAL), Systematic review, Target organ damage, Urinary biomarkers, Uromodulin (uUMOD)

## Abstract

**Purpose of Review:**

The growing burden of cardiovascular diseases has become a significant concern in both adult and youth populations. Urinary biomarkers of kidney function could provide useful insights that may aid in the early identification of individuals at higher risk of adverse cardiovascular outcomes. This systematic review aimed to assess associations between urinary biomarkers of kidney function and different measures of cardiovascular health.

**Recent Findings:**

PubMed, Scopus, and EBSCOhost were searched for articles published between January 2018 and December 2023. Studies exploring associations between urinary kidney biomarkers (alpha-1 microglobulin (uA1M), neutrophil gelatinase-associated lipocalin (uNGAL), uromodulin (uUMOD) and CKD273 classifier) and measures of cardiovascular health (blood pressure and markers of target organ damage) were included. We identified 1186 articles, with 22 studies eligible for inclusion. Among 12 studies reporting associations between uA1M and measures of cardiovascular health, six studies indicated positive associations with office blood pressure and three studies observed associations with different markers of target organ damage. Out of the nine studies that explored the link between uUMOD and cardiovascular health parameters, four found negative associations between uUMOD and blood pressure. With regard to uNGAL, only two out of the seven studies analysed reported varying associations with blood pressure, while neither of the two studies focusing on CKD273 observed any statistically significant results.

**Summary:**

Biomarkers of kidney tubule function, represented by uA1M and uUMOD, are relevant in the setting of cardiovascular health and should be assessed for utilisation in clinical practice to identify adverse cardiovascular outcomes at an early stage allowing for timely intervention.

**Graphical Abstract:**

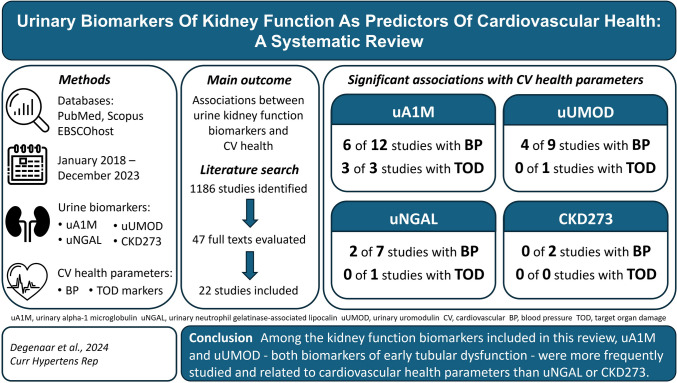

**Supplementary Information:**

The online version contains supplementary material available at 10.1007/s11906-025-01328-5.

## Introduction

Globally, non-communicable diseases (NCDs) have emerged as the predominant public health challenge, accounting for almost 74% of deaths annually [1, 2]. The burden of NCDs and particularly cardiovascular diseases (CVDs) is a growing concern in sub-Saharan African countries [3–5]. Approximately 80% of CVD-related deaths occur in low- and middle-income countries [6, 7]. Furthermore, the incidence and prevalence of CVDs are significantly increasing among youth [6, 8–10]. A great need exists for effective strategies and measures for the prevention and management of cardiovascular health in younger populations [3, 7, 8, 10].

Various cardiovascular risks, including high blood pressure, atherosclerosis, retinal microvascular and cardiac abnormalities, have been closely linked to kidney function [11–19]. Most of these associations have only been studied by using estimated glomerular filtration rate (eGFR) and urinary albumin-to-creatinine ratio (uACR) as clinical measures of kidney function [12, 16–19]. Both eGFR and uACR have several limitations with regard to sensitivity and specificity for evaluating early deterioration in kidney function [20]. Since eGFR and uACR mainly reflect glomerular function and injury, these traditional biomarkers incompletely capture kidney health at other parts of the nephron and not necessarily in the early development of kidney damage [20–22]. Several inexpensive, non-invasive and preclinical biomarkers of kidney function and injury have been identified which can indicate tubulointerstitial damage, inflammation, repair and fibrosis at early stages of disease development and progression [23–29]. Some of these urinary biomarkers include alpha-1 microglobulin (uA1M), neutrophil gelatinase-associated lipocalin (uNGAL), uromodulin (uUMOD) and the CKD273 proteomics classifier [23–29]. Identifying early subcellular and structural alterations instead of established irreversible kidney damage may provide comprehensive information on cardiovascular and kidney health, independent of glomerular dysfunction (eGFR) and/or damage (uACR) [23]. Given that different urinary kidney function biomarkers pose great value as promising risk predictors, it is important to investigate these biomarkers and how they associate with different measures of cardiovascular health. To the best of our knowledge, there is currently no systematic review that documents this information. Therefore, the aim of this systematic review was to investigate associations of different urinary biomarkers of kidney function (uA1M, uNGAL, uUMOD and CKD273) with measures of cardiovascular health, including blood pressure and markers of target organ damage (retinal microvascular parameters, arterial stiffness, atherosclerosis and measures of cardiac structure and function).

## Methods

This systematic review was conducted in accordance with the Preferred Reporting Items for Systematic Review and Meta-Analyses guidelines [30].

### Search Strategy

We conducted a literature search in the following databases: PubMed, Scopus (Elsevier) and EBSCOhost (Academic Search Complete, CINAHL, E-journals and Health Source: Nursing/Academic Edition) for articles published from 1 January 2018 until 31 December 2023). The full search strategies, with comprehensive search terms, can be seen in Supplementary Table 1 (Online Resource 1). References from relevant articles and reviews were also screened for potential articles meeting the inclusion criteria.

### Screening and Selection

The search results were screened for duplicates which were removed, followed by title and abstract screening; screening was performed independently by two reviewers (AD and OA) using the software Rayyan. Throughout the screening process Rayyan’s blinded mode was used to ensure that the reviewers’ decisions were not influenced by each other’s assessments. Full texts of eligible articles were then retrieved and independently assessed by two reviewers (AD and OA) to determine eligibility according to the inclusion/exclusion criteria. If a full text was unavailable, the corresponding author was contacted to gain access to the article, allowing two weeks for a response.

### Study Selection

We included the following study designs: randomised control trials, cohort studies and cross-sectional, observational and prospective studies. Given that urine samples are easily accessible, that urinary biomarkers can be analysed relatively cost-effectively and that they are sensitive to early stages of disease progression and organ damage [31, 32], we excluded studies reporting on biomarkers measured in plasma, serum and other bodily fluids. Letters, reviews, commentaries, case reports, abstracts, editorials, studies not written in English, animal, intra-uterine and genetic studies and studies focusing on pre-eclampsia or maternal hypertension were excluded as they fell outside the scope of this review.

Of interest were associations between different urinary biomarkers of kidney function (uA1M, uNGAL, uUMOD or CKD273 classifier) and blood pressure (twenty-four hour (24-h) ambulatory blood pressure, office brachial blood pressure or central blood pressure) or markers of target organ damage (retinal microvascular parameters, arterial stiffness, atherosclerosis and measures of cardiac structure and function including, but not limited to, left ventricular mass (LVM), left ventricular ejection fraction (LVEF) and markers indicative of systolic and diastolic dysfunction).

Any disagreements in title, abstract or full text screening and selection were resolved through comprehensive discussion among the two reviewers (AD and OA) until consensus was reached.

### Data Extraction

Relevant data from each individual article were extracted by one reviewer (AD). This included the first author’s name, year of publication, country, type of study design, study population, characteristics of the study population (age of participants, participant sex, mean/median blood pressure values, mean/median values of target organ damage markers as well as mean/median kidney function biomarker levels), main findings and any additional notes that were relevant to the findings reported.

### Quality and Bias Assessment

The methodological quality of cross-sectional and prospective studies was assessed by using appraisal tools from the Joanna Brigs Institute (JBI) and the modified Newcastle-Ottowa Scale (NOS). The JBI uses yes, no, or not applicable to evaluate eight different items: i) clear definition of inclusion sample; ii) description of study subjects and setting; iii) measurement of exposure; iv) objective and standard criteria used for measurements; v) identification of confounding factors; vi) strategies to confounding factors; vii) outcome measurements; and viii) appropriate statistical analysis. A higher score indicated a better study quality [33]. On the other hand, the NOS score is calculated based on three major domains: study participants (0–4 points), adjustment for confounding (0–2 points) and ascertainment of the exposure or outcome of interest (0–3 points), with a maximum score of 9 points representing the highest methodological quality [34].

To standardise the scoring and ensure comparability of studies with mixed methodologies, original scores from each tool were converted to a uniform 0–10 scale. Standardised scores were calculated and categorised into three quality levels: high (≥ 8), moderate (6–7) and low (< 6). High and moderate quality studies were included in this systematic review, while studies with low quality were excluded to mitigate the risk of bias.

## Results

### Search Results

**Fig. 1:**
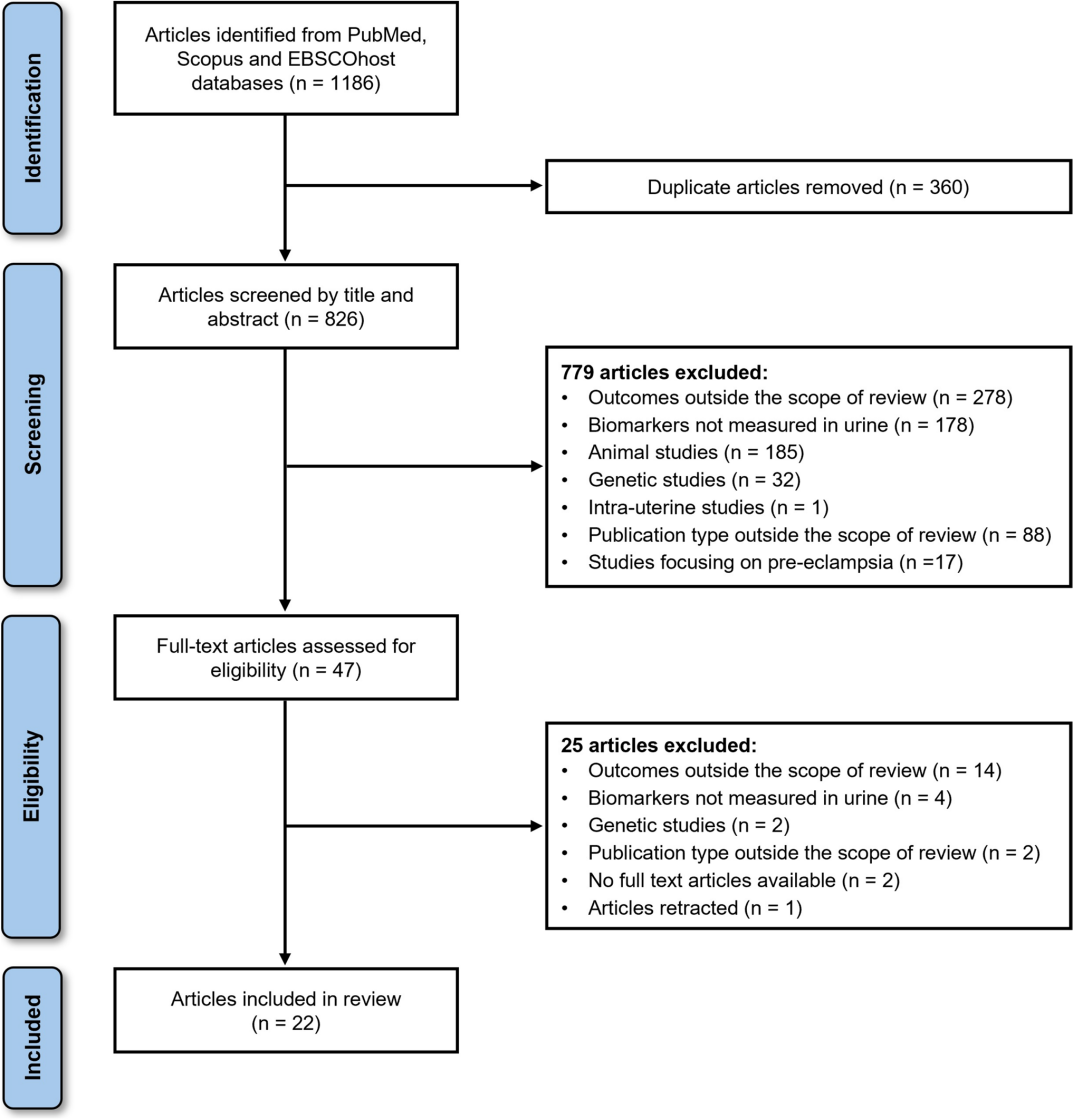
Preferred Reporting Items for Systematic Reviews and Meta-analyses flow chart of the study selection

A flow diagram of the selection process is depicted in Fig. 1. A total of 1186 potentially relevant articles were identified from PubMed, Scopus, and EBSCOhost databases. After removal of duplicates (*n* = 360) using Rayyan software, 826 articles were available for title and abstract screening, after which 779 articles were excluded for reasons including: i) outcomes and publication types outside the scope of this review; ii) studies with biomarkers measured in serum, plasma or other bodily fluids; iii) animal, genetic and intra-uterine studies; and iv) studies focusing on pre-eclampsia or maternal hypertension. Among the 47 articles selected for full-text screening, 25 were excluded as they did not report outcomes of interest for this review (*n* = 14), did not measure kidney function biomarkers in urine (*n* = 4), focused on genetics (*n* = 2), were publication types outside the scope of this review (*n* = 2), had no full-text articles available (*n* = 2) or the article was retracted (*n* = 1).

### Study Characteristics

The main characteristics of the studies reviewed are summarised in Table 1 and Table 2.

**Table 1:** Main characteristics of studies reporting associations between kidney biomarkers and blood pressure

Study; Year (ref)	Country	Study design	Study population	Biomarker(s)	Population characteristics	Main findings	Notes
Craig et al. 2022 [35]	South Africa	Cross-sectional study	Participants of the ExAMIN Youth SA study	A1M indexed to urinary creatinine	Black group*: • *N* = 526 • Age (years) = 7.46 ± 0.96 • Male (%) = 42 • SBP (mmHg) = 101 ± 10.0 • DBP (mmHg) = 65 ± 7.9 • MAP (mmHg) = 79.3 ± 7.9 • Urinary albumin (mg/l)^**†**^ = 6.28 (5.97; 6.53) • A1M/Cr (mmol/ g Cr)^**†**^ = 5.29 (5.16; 5.43) White group*: • *N* = 431 • Age (years) = 7.42 ± 0.84 • Male (%) = 52 • SBP (mmHg) = 103 ± 10.3 • DBP (mmHg) = 63 ± 7.0 • MAP (mmHg) = 79.2 ± 7.2 • Urinary albumin (mg/l)^**†**^ = 7.46 (7.16; 7.61) • A1M/Cr (mmol/ g Cr)^**†**^ = 4.72 (4.59; 4.84)	• A1M/Cr levels increased with each BP category in the Black group only (all *p* < 0.05) • In single, partial, and multiple regression: positive associations between A1M/Cr and SBP (β = 0.15; 95% CI, 0.06 to 0.19; *p* < 0.001), DBP (β = 0.18; 95% CI, 0.09 to 0.24; *p* < 0.001) and MAP (β = 0.11; 95% CI, 0.02 to 0.17; *p* = 0.018) in the Black group • In single, partial, and multiple regression: DBP associated positively with A1M/Cr in the White group (β = 0.10; 95% CI, 0.01 to 0.25; *p* = 0.038) • Multivariable adjusted binary logistic regression: likelihood of having elevated BP increased with 28% with each SD increase in A1M/Cr in the Black group (Odds ratio = 1.28; 95% CI, 1.10 to 1.50; *p* = 0.002), while no significance was reached in the White group	• Hypertension could not be confirmed, as BP was only measured on a single day
Ikeme et al. 2022 [36]	North America	Cross-sectional study	Participants of the SPRINT trial with a baseline SBP > 130 mmHg and eGFR < 60 ml/min/1.73m^2^	A1M NGAL UMOD	Total study population*: • *N* = 2436 • Age (years) = 73 ± 9 • Male (%) = 60 • SBP (mmHg) = 140 ± 16 • DBP (mmHg) = 74 ± 12 • eGFR (ml/min/1.73m^2^) = 46 ± 10 • uACR (mg/g)^**#**^ = 15 (7–48) • A1M (mg/ml)^**#**^ = 13 (7–25) • NGAL (ng/ml)^**#**^ = 28 (15–59) • UMOD (mg/ml)^**#**^ = 6.52 (4.26–9.93)	• Each SD higher SBP at baseline (16 mmHg) was associated with higher levels of A1M (10%) and lower levels of UMOD (−2%); all *p* < 0.05 • No association between baseline SBP and NGAL	• Associations were estimated using the MSG-LASSO method for variable selection • The authors reported standardised regression coefficients as percentages to facilitate comparison of effect sizes • All clinical and demographic characteristics were included as predictors of each urine biomarker • Authors controlled for urinary creatinine in all analyses to account for urine tonicity
Khan et al. 2023 [37]	North America	Prospective cohort study; follow-up median 9.9 years	Participants of the CARDIA study without hypertension, cardiovascular or kidney disease at baseline	A1M UMOD	Total study population^**#**^: Baseline: • *N* = 1170 • Age (years) = 45 (42–48) • Male (%) = 44 • SBP (mmHg) = 109 (103–116) • DBP (mmHg) = 67 (61–72) • eGFR (ml/min/1.73m^2^) = 111 (104–117) • uACR (mg/g) = 4.1 (2.8–6.0) Black participants: • N = 463 • Age (years) = 43 (40–47) • Male (%) = 43 • SBP (mmHg) = 111 (105–117) • DBP (mmHg) = 68 (64–73) • eGFR (ml/min/1.73m^2^) = 113 (106–119) • uACR (mg/g) = 4.2 (2.8–6.6) White participants: • *N* = 707 • Age (years) = 46 (43–48) • Male (%) = 45 • SBP (mmHg) = 108 (102–115) • DBP (mmHg) = 66 (60–71) • eGFR (ml/min/1.73m^2^) = 110 (103–116) • uACR (mg/g) = 4.0 (2.8–5.6) Follow-up: • 32% of the participants developed incident hypertension • 47% of Black and 23%of White participants developed hypertension • After 10 years, SBP and DBP increased by an average of 9.6 and 5.6 mmHg, respectively, in Black adults and an average of 5.1 and 3.6 mmHg, respectively, in White adults	• No statistically significant associations of A1M or UMOD with incident hypertension • No statistically significant associations of A1M or UMOD with 10-year change in SBP or DBP	• Authors adjusted for urinary creatinine in multiple regression models to account for urine tonicity • Full adjustments additionally included smoking, diabetes, SBP, DBP, lipids, body mass index, physical activity, education, income, eGFR and urinary albumin • The associations of biomarker concentrations with incident hypertension were explored by using interval-censored proportional hazards regression models • The association of biomarkers with BP changes over time were investigated with linear mixed models
Seeman et al., 2018 [38]	Czech Republic	Cross-sectional study	Children with ADPKD	A1M indexed to urinary creatinine	Total study population^**#**^: • *N* = 37 • Age (years) = 11.2 (2–18) • Male (%) = 54.1 • Hypertension (%) = 46 • eGFR (ml/min/1.73m^2^) = 120 (67–197) • Albuminuria (mg/mmol Cr) = 2.54 (0.54–37.3) • Pathological albuminuria (%) = 49 • A1M/Cr (mg/mmol) = 3.22 (0.04–10.2) • Pathological A1M/Cr (%) = 65	• No significant correlation was found between A1M/Cr and office blood pressure	• Pathological uACR was defined as urinary albumin excretion > 2.2 mg/mmol Cr • Pathological A1M/Cr was defined as A1M/Cr > 0.55 mg/mmol • Correlations between BP index and A1M was tested by Spearman correlation coefficient and the authors did not explore associations with multiple regression analysis
Ishiwata et al*.* 2021 [39]	Japan	Prospective study (median follow-up: 488 days)	Hospitalised patients with acute heart failure	A1M indexed to urinary creatinine	Total study population*: • *N* = 623 • Age (years) = 74 ± 13 • Male (%) = 62.4 • A1M/Cr (mg/g Cr)^**#**^ = 12.9 (5.92–30.7) Quartile 1: 0.50 ≤ A1M/Cr (mg/g Cr) ≤ 5.91 • N = 156 • Age (years) = 72 ± 14 • Male (%) = 62.8 • SBP (mmHg) = 131 ± 27 • DBP (mmHg) = 80 ± 21 • LVEF (%)^**#**^ = 47 (31–62) • History of hypertension (%) = 44.4 • eGFR (ml/min/1.73m^2^) = 79.8 (30.5) Quartile 2: 5.92 ≤ A1M/Cr (mg/g Cr) ≤ 12.9 • *N* = 156 • Age (years) = 74 ± 13 • Male (%) = 67.9 • SBP (mmHg) = 131 ± 26 • DBP (mmHg) = 78 ± 20 • LVEF (%)^**#**^ = 44 (31–63) • History of hypertension (%) = 48.4 • eGFR (ml/min/1.73m^2^) = 68.7 (28.3) Quartile 3: 13.0 ≤ A1M/Cr (mg/g Cr) ≤ 30.6 • *N* = 155 • Age (years) = 76 ± 13 • Male (%) = 62.6 • SBP (mmHg) = 137 ± 27 • DBP (mmHg) = 79 ± 21 • LVEF (%)^**#**^ = 50.5 (36–63) • History of hypertension (%) = 58.4 • eGFR (ml/min/1.73m^2^) = 65.0 (35.4) Quartile 4: 30.7 ≤ A1M/Cr (mg/g Cr) ≤ 376.9 • *N* = 156 • Age (years) = 77 ± 11 • Male (%) = 56.4 • SBP (mmHg) = 138 ± 27 • DBP (mmHg) = 76 ± 21 • LVEF (%)^**#**^ = 55.5 (43–65) • History of hypertension (%) = 64.3 • eGFR (ml/min/1.73m^2^) = 47.3 (34.9)	• Ascending quartiles of A1M/Cr: individuals in the highest A1M group were more likely to have higher SBP (*p* = 0.013) and a history of hypertension (*p* = 0.002)	• The relationship between characteristics and quartiles of A1M/Cr were compared cross-sectionally (at baseline) by using a one-way analysis of variance test, Kruskal–Wallis test or chi-squared test where appropriate • Authors did not explore associations between A1M, SBP or history of hypertension with multiple regression analyses
Sun et al*.* 2020 [40]	China	Prospective cohort study (10-year follow-up)	Participants with prediabetes and without kidney damage in the INDEED study	A1M	Total study population*: • *N* = 1451 • Age (years) = 51.4 ± 8.8 • Male (%) = 81.7 Moderate stable group: • *N* = 726 • Age (years) = 51.3 ± 8.6 • Male (%) = 83.2 • SBP (mmHg) = 133 ± 12.7 • DBP (mmHg) = 86 ± 9.2 Ten-year outcomes: • eGFR (ml/min/1.73m^2^) = 101 ± 12.8 • uACR (mg/mmol)^**#**^ = 1.6 (0.8–3.5) • A1M(mg/l)^**#**^ = 16.6 (7.5–30.0) Low stable group: • *N* = 323 • Age (years) = 47.3 ± 8.8 • Male (%) = 76.8 • SBP (mmHg) = 115 ± 10.8 • DBP (mmHg) = 77 ± 8.0 Ten-year outcomes: • eGFR (ml/min/1.73m^2^) = 103 ± 13.6 • uACR (mg/mmol)^**#**^ = 1.2 (0.7–2.3) • A1M (mg/l)^**#**^ = 13.7 (6.0–25.7) Moderate-increasing group: • *N* = 176 • Age (years) = 55.1 ± 7.1 • Male (%) = 84.1 • SBP (mmHg) = 146 ± 14.5 • DBP (mmHg) = 92 ± 9.3 Ten-year outcomes: • eGFR (ml/min/1.73m^2^) = 94.4 ± 13.4 • uACR (mg/mmol)^**#**^ = 1.9 (0.9–5.7) • A1M (mg/l)^**#**^ = 16.0 (7.4–32.8) Moderate-decreasing group: • *N* = 181 • Age (years) = 54.2 ± 8.6 • Male (%) = 81.8 • SBP (mmHg) = 159 ± 13.7 • DBP (mmHg) = 98 ± 11.9 Ten-year outcomes: • eGFR (ml/min/1.73m^2^) = 95.7 ± 14.6 • uACR (mg/mmol)^**#**^ = 2.1 (1.1–5.3) • A1M (mg/l)^**#**^ = 16.6 (7.9–30.8) High-stable group*: • *N* = 45 • Age (years) = 57.0 ± 6.8 • Male (%) = 84.4 • SBP (mmHg) = 171 ± 19.8 • DBP (mmHg) = 99 ± 11.1 Ten-year outcomes: • eGFR (ml/min/1.73m^2^) = 89.4 ± 14.3 • uACR (mg/mmol)^**#**^ = 2.9 (1.4–8.1) • A1M (mg/l)^**#**^ = 26.8 (14.2–39.8)	• Compared with the low-stable group, A1M increased among the other four groups (all *p* < 0.01) • Linear regression analysis: high stable SBP group were associated with higher levels of A1M (β = 0.22; *p* < 0.05) • Logistic regression analyses: significant associations between the high stable SBP group and A1M > 29.75 mg/L (Odds ratio = 2.91; 95% CI, 1.43 to 5.94; *p* < 0.05)	• During the 10-year follow-up, five SBP trajectories were distinctly separated among the 1451 participants with three or more SBP measurements • Linear regression, binary logistic regression and multinomial logistic regression models were used to assess the association between SBP trajectory groups and indicators of early kidney damage • Authors did not include urinary creatinine in any of the analyses • Variables included in the final multivariable regression model were age, gender, history of myocardial infarction, stroke, cancer, education, physics, smoking status, drinking status, salt habit, triglyceride, total cholesterol, body mass index, LDL, HDL, hsCRP, antihypertensive, hypoglycemic and lipid-lowering drugs
Biliotti et al. 2020 [41]	Italy	Prospective study (follow-up 6 months after the end of antiviral therapy)	Child–Pugh A cirrhotic patients receiving direct-acting antiviral treatment for chronic hepatitis C	A1M indexed to urinary creatinine	Total study population*: • *N* = 135 • Age (years) = 62.6 ± 10.8 • Male(%) = 61.5 • Arterial hypertension (%) = 41.5 • Combined diabetes and arterial hypertension (%) = 11.9 • eGFR (ml/min/1.73m^2^) = 89.7 ± 16.1 • A1M/Cr (µg/mg)^**#**^ = 21 (17.3–27.5) • Tubular dysfunction (%) = 23.7 No proximal tubular dysfunction: • *N* = 103 • Age (years)^**#**^ = 61 (53–69) • Male (%) = 63.1 • Arterial hypertension (%) = 34.9 • Combined diabetes and arterial hypertension (%) = 7.8 • eGFR (ml/min/1.73m^2^)^**#**^ = 93.0 (84.0–102) • uACR ≥ 30 mg/g (%) = 9.7 Proximal tubular dysfunction: • *N* = 32 • Age (years)^**#**^ = 66 (59.2–75.7) • Male (%) = 56.2 • Arterial hypertension (%) = 62.5 • Combined diabetes and arterial hypertension (%) = 25 • eGFR (ml/min/1.73m^2^)^**#**^ = 84.5 (74.5–97.2) • uACR ≥ 30 mg/g (%) = 37.5	• Compared with patients without tubular dysfunction, those with tubular dysfunction had a more frequent history of arterial hypertension (*p* = 0.006) and combined diabetes and arterial hypertension (*p* = 0.008) • In multiple logistic regression analyses the presence of diabetes together with arterial hypertension were associated with tubular dysfunction (Odds ratio = 4.23; 95% CI, 1.10 to 16.27; *p* < 0.05)	• Tubular dysfunction was defined by urinary A1M/Cr values higher than 14 μg/mg • A cross-sectional analysis was performed to investigate the relationship between independent predictors and proximal tubular dysfunction at baseline
Wang et al. 2019 [42]	China	Cross-sectional study	Elderly community participants (> 60 years of age)	A1M indexed to urinary creatinine	Total study population*: • *N* = 691 • Age (years) = 67.7 ± 6.1 • Male (%) = 42.1 • Hypertension (%) = 59.9 • eGFR (ml/min/1.73m^2^) = 76.9 ± 11.8 • A1M/Cr (median; mg/g) = 14.9 Normoalbuminuric group: • *N* = 482 • Age (years) = 67.5 ± 5.87 • Male (%) = 46.3 • SBP (mmHg) = 132 ± 17.5 • DBP (mmHg) = 82 ± 9.39 • Hypertension (%) = 55.2 • eGFR (ml/min/1.73m^2^) = 78.2 ± 10.9 • A1M/Cr (mg/g) = 12.2 (7.64–19.4) Albuminuric group: • *N* = 209 • Age (years) = 68.2 ± 9.49 • Male (%) = 32.5 • SBP (mmHg) = 138 ± 18.7 • DBP (mmHg) = 84 ± 10.9 • Hypertension (%) = 70.8 • eGFR (ml/min/1.73m^2^) = 73.8 ± 13.2 • A1M/Cr (mg/g)^**#**^ = 29.13 (15.8–55.6)	• The proportion of participants with increased A1M/Cr was 49.8%, 37.6% and 78.0% in the overall, normoalbuminuric and albuminuric groups, respectively • Among the normoalbuminuric participants, 39.9% and 47.2% of individuals with hypertension and hypertension with diabetes, respectively, had increased A1M/Cr • Hypertension (Odds ratio = 1.38; 95% CI, 1.02 to 1.93; *p* = 0.011) was a strong independent risk factor of increased A1M/Cr	• Normoalbuminuria was defined as a uACR < 30 mg/g in morning urine • Increased A1M/Cr was defined as A1M/Cr > 15 mg/g
Bakhoum et al. 2022 [43]	North America	Multicenter, prospective, observational study (median follow-up of 7 months)	CKiD participants	UMOD indexed to urinary creatinine	Total study population^**#**^: • *N* = 436 • Age (years) = 12.4 (9–15) • Male (%) = 59 • Clinic SBP (mmHg) = 107 (100–116) • 24-h SBP (mmHg) = 112 (104–119) • eGFR (ml/min/1.73m^2^) = 50 (36–62) • UMOD/Cr (mg/g Cr) = 0.12 (0.05–0.23) Quartile 1: UMOD/Cr (mg/g Cr) < 0.05 • *N* = 109 • Age (years) = 14.3 (12–16) • Male (%) = 60 • Hypertension (%) = 19 • Clinic SBP (mmHg) = 111 (103–118) • Clinic DBP (mmHg) = 68 (61–75) • 24-h SBP (mmHg) = 115 (108–122) • 24-h DBP (mmHg) = 67 (62–72) • eGFR (ml/min/1.73m^2^) = 53 (35–71) Quartile 2: 0.05 ≤ UMOD/Cr (mg/g Cr) < 0.12 • *N* = 109 • Age (years) = 13.9 (11–16) • Male (%) = 56 • Hypertension (%) = 20 • Clinic SBP (mmHg) = 108 (100–120) • Clinic DBP (mmHg) = 63 (57–73) • 24-h SBP (mmHg) = 113 (107–121) • 24-h DBP (mmHg) = 67 (61–73) • eGFR (ml/min/1.73m^2^) = 45 (31–61) Quartile 3: 0.12 ≤ UMOD/Cr (mg/g Cr) < 0.23 • *N* = 109 • Age (years) = 11.8 (9–15) • Male (%) = 60 • Hypertension (%) = 23 • Clinic SBP (mmHg) = 106 (98–116) • Clinic DBP (mmHg) = 63 (59–73) • 24-h SBP (mmHg) = 111 (103–118) • 24-h DBP (mmHg) = 66 (61–72) • eGFR (ml/min/1.73m^2^) = 49 (36–59) Quartile 4: UMOD/Cr (mg/g Cr) ≥ 0.23 • N = 109 • Age (years) = 8.8 (6–11) • Male (%) = 61 • Hypertension (%) = 24 • Clinic SBP (mmHg) = 104 (97–111) • Clinic DBP (mmHg) = 64 (59–71) • 24-h SBP (mmHg) = 109 (100–116) • 24-h DBP (mmHg) = 65 (61–71) eGFR (ml/min/1.73m^2^) = 51 (41–64)	• The lowest UMOD/Cr quartile (< 0.05 mg/g Cr) had the highest clinic SBP (*p* < 0.05) • The highest UMOD/Cr quartile (≥ 0.23 mg/g Cr) had the lowest 24-h SBP (*p* < 0.05) • Univariate: each twofold higher UMOD/Cr ratio was associated with 1.66 mmHg (95% CI, −2.31 to −1.00) lower 24-h SBP (*p* < 0.001) and 0.49 mmHg (95% CI, −0.97 to −0.01) lower 24-h DBP (*p* = 0.05) • Univariate: each twofold higher UMOD/Cr ratio was associated with a 1.71 mmHg (95% CI, −2.45 to −0.97) lower clinic SBP (*p* < 0.001) and a 0.90 mmHg (95% CI, −1.59 to −0.22) lower clinic DBP (*p* = 0.01) • Multivariate: UMOD/Cr ratio was not significantly associated with 24-h or clinic BP	• A cross-sectional analysis was performed to investigate the relationship between baseline UMOD/Cr and BP patterns • Inclusion of age in the multivariable models attenuated the association between UMOD/Cr and BP
Nqebele et al. 2019 [44]	South Africa	Case control study	Black South Africans with clinically diagnosed hypertension-attributed CKD with an eGFR ≤ 60 ml/min/1.73m^2^ (age ≥ 18 years at disease onset), first-degree relatives, and unrelated controls (geographically and ethnically matched healthy controls with normal kidney function, negative HIV serology and normal BP)	UMOD indexed to urinary creatinine	Hypertension-attributed CKD^**#**^: • *N* = 71 • Age (years) = 48 (41–53) • Male (%) = 65 • SBP (mmHg) = 141 (132–168) • DBP (mmHg) = 86 (78–97) • eGFR (ml/min/1.73m^2^) = 8 (4–12) • UMOD (ug/ml)* = −0.4 ± 1.9 • UMOD/Cr (ug/mg) = 0.6 (0.2–3.0) Unrelated controls^**#**^: • *N* = 58 • Age (years) = 41 (34–46) • Male (%) = 45 • SBP (mmHg) = 118 (113–130) • DBP (mmHg) = 73 (69–77) • eGFR (ml/min/1.73m^2^) = 121 (99–130) • UMOD (ug/ml)* = 1.1 ± 1.7 • UMOD/Cr (ug/mg) = 3 (1.0–7.0)	• In the hypertension -attributed CKD cases, significantly lower UMOD, UMOD/Cr as well as higher SBP and DBP were present compared to the controls (*p* < 0.001) • UMOD correlated negatively with SBP (*r* = −0.23; *p* = 0.004) • For each 1-SD increase in UMOD level, the multivariable-adjusted odds ratio for hypertension attributed CKD was 0.62 (95% CI, 0.48 to 0.81; *p* < 0.001)	• Authors did not include UMOD/Cr in any of the analyses
Steubl et al. 2020 [45]	North America	Observational, community-based cohort study	Participants from the Cardiovascular Health Study (men and women ≥ 65 years)	UMOD	Total study population*: • *N* = 933 • Age (years) = 78.1 ± 4.8 • Male (%) = 39.7 • SBP (mmHg) = 137.0 ± 21.0 • DBP (mmHg) = 69.8 ± 11.0 • Hypertension (%) = 60 • UMOD (ng/ml) = 30 500 ± 19,800 • eGFR (ml/min/1.73m^2^) = 63.4 ± 18.6 • eGFR < 60 ml/min/1.73m^2^ (%) = 41.7 • uACR (mg/g) = 3.8 ± 1.9 • uACR > 30 mg/g (%) = 20.5	• In univariateand multivariate (adjusted for demographics, CVD risk factors, prevalent CVD, inflammatory variables, eGFR and albuminuria) analysis, hypertension (β = −4.30; 95% CI, −7.50 to −1.10) was associated with lower uUMOD (*p* < 0.05)	• Authors did not include urinary creatinine in any of the analyses
Josipović et al. 2023 [46]	Croatia	Cross-sectional study	Young, middle-aged apparently healthy individuals	UMOD indexed to urinary creatinine	Total study population*: • *N* = 326 • Age (years) = 36 ± 8 • Male (%) = 49.4 Optimal BP group: • N = 103 • Age (years)^**#**^ = 36 (30–42) • Male (%) = 71.8 • Office brachial SBP (mmHg) = 108 ± 7.1 • Office brachial DBP (mmHg) = 71 ± 6.2 • eGFR (ml/min/1.73m^2^)^**#**^ = 107 (96.3–115) • uACR (mg/g Cr)^**#**^ = 4.14 (2.64–6.05) • A1M/Cr (mg/g Cr)^**#**^ • = 5.6 (3.3–7.7) • UMOD/Cr (mg/g Cr)^**#**^ = 43 (27–66) Subgroup of optimal BP: • *N* = 47 • 24-h SBP (mmHg) = 118 ± 7.72 • 24-h DBP (mmHg) = 72 ± 5.83 • 24-h PP (mmHg) = 45 ± 5.5 • Daytime SBP (mmHg) = 121 ± 7.93 • Daytime DBP (mmHg) = 75 ± 6.22 • Daytime PP (mmHg) = 45 ± 5.7 • Nighttime SBP (mmHg) = 109 ± 9.45 • Nighttime DBP (mmHg) = 64 ± 6.89 • Nighttime PP (mmHg) = 44 ± 5.56 Prehypertension: • *N* = 140 • Age (years)^**#**^ = 37 (29–44) • Male (%) = 42.7 • Office brachial SBP (mmHg) = 127 ± 80.8 • Office brachial DBP (mmHg) = 81 ± 6.5 • eGFR (ml/min/1.73m^2^)^**#**^ = 105 (91–113) • uACR (mg/g Cr)^**#**^ = 4.1 (2.3–6.6) • A1M/Cr (mg/g Cr)^**#**^ = 4.3 (3.2–6.7) • UMOD/Cr (mg/g Cr)^**#**^ = 42.9 (25.5–65.3) Subgroup of prehypertension: • *N* = 68 • 24-h SBP (mmHg) = 131 ± 10.8 • 24-h DBP (mmHg) = 79 ± 6.73 • 24-h PP (mmHg) = 51 ± 10.4 • Daytime SBP (mmHg) = 134 ± 10.5 • Daytime DBP (mmHg) = 82 ± 6.89 • Daytime PP (mmHg) = 51.8 ± 10.5 • Nighttime SBP (mmHg) = 121 ± 13.1 • Nighttime DBP (mmHg) = 70 ± 8.27 • Nighttime PP (mmHg) = 51 ± 11.4 Hypertension: • *N* = 80 • Age (years)^**#**^ = 39 (33–45) • Male (%) = 37.5 • Office brachial SBP (mmHg) = 144 ± 94.2 • Office brachial DBP (mmHg) = 94 ± 8.2 • eGFR (ml/min/1.73m^2^)^**#**^ = 105 (94–110) • uACR (mg/g Cr)^**#**^ = 4.7 (2.8–8.3) • A1M/Cr (mg/g Cr)^**#**^ = 5.1 (3.6–8.4) • UMOD/Cr (mg/g Cr)^**#**^ = 40.6 (24.1–60.4) Subgroup of hypertension: • *N* = 60 • 24-h SBP (mmHg) = 138 ± 15.2 • 24-h DBP (mmHg) = 89 ± 11.1 • 24-h PP (mmHg) = 48 ± 8.61 • Daytime SBP (mmHg) = 140 ± 16.1 • Daytime DBP (mmHg) = 92 ± 11.0 • Daytime PP (mmHg) = 50 ± 11.3 • Nighttime SBP (mmHg) = 127 ± 18.5 • Nighttime DBP (mmHg) = 82 ± 12.7 Nighttime PP (mmHg) = 47 ± 10.1	• No significant differences in UMOD/Cr between BP groups • UMOD/Cr correlated positively with daytime SBP (Rho 0.32; *p* = 0.04) in the optimal BP group • UMOD/Cr correlated negatively with 24-h PP (Rho −0.28; *p* = 0.04) and nighttime PP (Rho −0.31; *p* = 0.02) in the prehypertension group • UMOD/Cr correlated negatively with 24-h DBP (Rho −0.29; *p* = 0.04), daytime DBP (Rho −0.27; *p* = 0.04) and nighttime SBP (Rho −0.29; *p* = 0.04) in the hypertension group	• 24-h ABPM was recorded in a subgroup of 175 individuals • Correlations between ABPM parameters and UMOD/Cr were evaluated by Spearman’s correlation coefficients and the authors did not explore associations in multiple regression analysis
Degenaar et al*.* 2023 [47]	South Africa	Cross-sectional study	Apparently healthy adults with no history of CVD from the African-PREDICT study	UMOD indexed to urinary creatinine CKD273 classifier	Total study population*: • *N* = 956 • Age (years) = 24.5 ± 3.12 • Male (%) = 51.2 • 24-h SBP (mmHg) = 117 ± 9.58 • 24-h DBP (mmHg) = 69 ± 5.96 • Office SBP (mmHg) = 123 ± 10.5 • Office DBP (mmHg) = 73 ± 7.87 • eGFR (ml/min/1.73m2) • = 143 ± 21.9 • Urinary albumin (mg/l)^**†**^ = 5.43 (5.21; 5.65) • UMOD/Cr (mg/g Cr)^**†**^ = 40.9 (18.9; 81.3) • CKD273 classifier = −0.55 ± 0.39	• Higher 24-h and office SBP were present in the lower 25th percentile of UMOD/Cr compared to the upper 25th percentile (all *p* < 0.05) • Higher 24-h DBP and office BP were present in the upper 25th percentile of the CKD273 classifier compared to the lower 25th percentile (all *p* < 0.05) • Multiple regression: no significant associations of UMOD/Cr and CKD273 classifier with office SBP in the total group	• The lower 25th percentile of UMOD/Cr and the upper 25th percentile of the CKD273 classifier represented more unfavourable kidney function groups • Authors did not explore associations of 24-h BP and office DBP with UMOD/Cr and CKD273 classifier in multiple regression analyses
Muiru et al. 2019 [48]	North America	Cross-sectional study	HIV-positive participants in the MACS and the WIHS studies	A1M NGAL UMOD	Total study population^**#**^: • *N* = 198 • Age (years) = (41–54) • Male (%) = 44 • SBP (mmHg) = 126 (114–137) • DBP (mmHg) = 77 (71–86) • Hypertension (%) = 48 • eGFR (ml/min/1.73m^2^) = 103 (88–116) • uACR (mg/g) = 3.2 (1.9–7.1) • uACR > 30 mg/g (%) = 8	• In multivariable adjustment in simultaneous linear equations and after MSG-LASSO selection: no significant associations of A1M, NGAL or UMOD with hypertension	• Authors evaluated associations of risk factors with biomarker levels in i) separate unadjusted linear regression models; ii) multivariable simultaneous linear equations; and iii) MSG-LASSO selection • Authors controlled for urinary creatinine in all analyses to account for urine tonicity
Greenberg et al. 2018 [49]	North America	Prospective cohort study (median 5.4 years of follow-up)	Children with congenital heart disease enrolled in the TRIBE-AKI study	NGAL	Total study population at five-year follow up^**#**^: • N = 110 • Baseline age (years)* = 3.73 ± 3.9 • Male (%) = 52 Biomarker level at year five: • eGFR (ml/min/1.73m^2^) = 113 (103–126) • uACR (mg/g) = 5.1 (2.8–12.6) • NGAL (ng/ml) = 4.4 (2.1–10.2) Group with hypertension at year five: • *N* = 20 • NGAL (year 5; ng/ml) = 7.07 (2.1–32.2) • NGAL elevated at year five (n; %) = 2 (10) • NGAL (change from baseline; ng/ml) = 2.7 (−2.5–39.4) Group without hypertension at year five: • *N* = 89 • NGAL (year 5; ng/ml) = 4.3 (2.2–8.4) • NGAL elevated at year five (n, %) = 1 (1) • NGAL (change from baseline; ng/ml) = 0.8 (−3.3–4.2)	• Five-year median NGAL was not significantly higher in children with vs without hypertension • There was no significant difference in absolute change in the NGAL concentrations from baseline to five-years in patients with vs without hypertension • Hypertension was associated with a higher proportion of patients with five-year NGAL concentrations above normal for age (*p* = 0.03) • The lack of associations of NGAL with hypertension remained unaffected when adjusting five-year NGAL concentrations for urinary creatinine	• A cross-sectional analysis was performed to investigate the association between five-year NGAL and hypertension • The 95th percentile biomarker cutoffs by age and gender were used as abnormal thresholds for NGAL • Authors did not explore associations between hypertension and NGAL in multiple regression analyses
Mamilly et al. 2021 [50]	North America	Cross-sectional study	Patients between the ages of 10 and 21 years with type-1 diabetes mellitus for more than 1 year as well as controls	NGAL indexed to urinary creatinine	Patients^**#**^: • *N* = 21 • Age (years) = 16.8 (15–19) • Male (%) = 38 • ABPM SBP (mmHg) = 107 (103–111) • ABPM DBP (mmHg) = 66 (63–68) • Daytime SBP (mmHg) = 109 (105–116) • Daytime DBP (mmHg) = 68 (65–73) • Nighttime SBP (mmHg) = 99 (95–103) • Nighttime DBP (mmHg) = 57 (55–59.2) • SBP dipping (mmHg) = 9.9 (6.2–14.4) • DBP dipping (mmHg) = 16.6 (12.4–22.4) • uACR (ug/mg) = 14.7 (9.8–20.8) • NGAL/Cr (ng/mg) = 34.2 (15.4–84.2) Controls: • *N* = 10 • Age (years) = 14.5 (12–15) • Male (%) = 50 • uACR (ug/mg) = 0.7 (0.6–0.9) • NGAL/Cr (ng/mg) = 3.7 (3.2–6.3)	• No differences in NGAL/Cr were found between cases with normal vs abnormal dipping of SBP • NGAL/Cr negatively correlated with nocturnal SBP dipping (*r* = −0.47; 95%CI, −0.76 to −0.03; *p* < 0.05)	• Nocturnal dipping of 10% or more was considered normal whereas dipping of less than 10% was considered abnormal • Spearman’s correlation was used for summarising the relationship of ABPM patterns with NGAL/Cr and the authors did not explore associations in multiple regression analysis
Hosohata et al. 2020 [51]	Japan	Cross-sectional study	Hypertensive patients	NGAL indexed to urinary creatinine	Total study population*: • *N* = 147 • Age (years) = 72.8 ± 8.3 • Male (%) = 60.5 • SBP (mmHg) = 133 ± 14.2 • DBP (mmHg) = 74 ± 11.0 • eGFR (ml/min/1.73m^2^)^**#**^ = 60.2 (42.3–70.9) • uACR ≥ 30 mg/g Cr (n, %) = 62 (42.2) • NGAL/Cr (ng/mg Cr) = 13.0 ± 12.0 Group with lower eGFR (< 60 ml/min/1.73m^2^): • *N* = 73 • Age (years) = 73.0 ± 9.3 • Male (%) = 64.4 • SBP (mmHg) = 133 ± 14.6 • DBP (mmHg) = 75 ± 12.6 • uACR ≥ 30 mg/g Cr (n, %) = 40 (54.8) • NGAL/Cr (ng/mg Cr) = 13.9 ± 13.0 Group with higher eGFR (≥ 60 ml/min/1.73m^2^): • *N* = 74 • Age (years) = 72.8 ± 7.3 • Male (%) = 56.8 • SBP (mmHg) = 133 ± 13.9 • DBP (mmHg) = 74 ± 9.2 • uACR ≥ 30 mg/g Cr (n, %) = 22 (29.7) • NGAL/Cr (ng/mg Cr) = 12.2 ± 10.9	• When stratified according to eGFR values (< 60 ml/min/1.73m^2^ and ≥ 60 ml/min/1.73m^2^): no significant correlations of NGAL/Cr with SBP or DBP were found in either of the groups	• Authors only used Spearman’s rank method to determine correlations between BP and NGAL/Cr and did not explore associations with multiple regression analyses
Tsingos et al. 2019 [52]	Switzer land	Cross-sectional study	ADPKD patients and age and gender matched controls (aged < 18 years)	NGAL	ADPKD patients*: • *N* = 15 • Age (years) = 10.2 ± 4.3 • Male (%) = 46.7 • SBP > 90th percentile (n, %) = 2 (13.3) • DBP > 90th percentile (n, %) = 2 (13.3) • NGAL (ng/ml) = 26.4 ± 43.5 Controls*: • *N* = 15 • Age (years) = 10.4 ± 3.4 • Male (%) = 46.7 • SBP > 90th percentile (n, %) = 0 (0) • DBP > 90th percentile (n, %) = 3 (20) • NGAL (ng/ml) = 27.2 ± 52.8	• No differences in NGAL levels were observed between the ADPKD patients and control groups • Correlation analyses in the patient group: no association between NGAL and MAP	• Correlations between BP and NGAL were done using Pearson’s correlations and the authors did not explore associations in multiple regression analysis • Authors did not include urinary creatinine in any of the analyses
Wallbach et al. 2018 [53]	Germany	Prospective study (follow-up 6 months)	Patients with resistant hypertension undergoing baroreflex activation therapy	CKD273	Total study population*: • *N* = 32 • Age (years) = 56 ± 11 • Male (%) = 47 Baseline: • ABPM SBP (mmHg) = 146 ± 16.1 • Office SBP (mmHg) • = 170 ± 25 • Office DBP (mmHg) = 90 ± 18 • eGFR (ml/min/1.73m^2^) = 76 ± 30 • uACR (mg/g)^**#**^ = 25.5 (12.5–175) • CKD273 = −0.02 ± 0.53 Follow up: • ABPM SBP (mmHg) = 141 ± 20.9 • Office SBP (mmHg) = 149 ± 29 • Office DBP (mmHg) = 82 ± 18 • eGFR (ml/min/1.73m^2^) = 76 ± 30 • uACR (mg/g)^**#**^ = 25.7 (11.4–69.3) • CKD273 = −0.06 ± 0.56	• After six months of baroreflex activation therapy there was no significant change in CKD273 score • Correlation analysis of the changes between baseline and after six months of baroreflex activation therapy: moderate correlation between changes in 24-h SBP values and changes on the CKD273 score (without reaching statistical significance: *r* = 0.18; *p* = 0.315) • By stratification of the data regarding 24-h ambulatory BP response, there was a statistically significant (*p* = 0.011) reduction in the CKD273 score from a mean of 0.16 (95% CI, −0.16 to 0.56) to −0.44 (95% CI, −0.63 to 0.05) after baroreflex activation therapy in patients with systolic ambulatory BP decrease of ≥ 5 mmHg	• Participants were classified as responders if there was a decrease of ≥ 5 mm Hg in 24-h ambulatory SBP • Authors did not explore associations between BP and CKD273 with multiple regression analyses • The differences between baseline and month six variables were analysed by nonparametric Wilcoxon tests or paired two-sided t tests (without correction for multiple testing) • Differences between the responders and non-responders were calculated with Mann–Whitney tests

*Values expressed as mean ± standard deviation; ^**#**^ values expressed as median (Interquartile range), ^**†**^ values expressed as geometric mean (5th and 95th percentile) or frequency and percentage. Abbreviations: 24-h, twenty-four hour; *A1M*, alpha-1 microglobulin; *A1M/Cr*, alpha-1 microglobulin-to-creatinine ratio; *ABPM*, ambulatory blood pressure monitoring; *ADPKD*, autosomal dominant polycystic kidney disease; *BP*, blood pressure; *CARDIA*, Coronary Artery Risk Development in Young Adults; *CKiD*, Chronic Kidney Disease in Children; *CI*, confidence interval; *DBP*, diastolic blood pressure; *eGFR*, estimated glomerular filtration rate; *ExAMIN Youth SA*, Exercise, Arterial Modulation and Nutrition in Youth South Africa; *HDL*, high-density lipoprotein; *HIV*, human immunodeficiency virus; *hsCRP*, high sensitivity C reactive protein; *INDEED*, Incidence, Development, and Prognosis of Diabetic Kidney Disease; *LDL*, low-density lipoprotein; *LVEF*, left ventricular ejection fraction; *MACS*, Multicenter *AIDS* Cohort Study; *MAP*, mean arterial pressure; *MSG -LASSO*, multivariable sparse group least absolute shrinkage and selection operator; *NGAL*, neutrophil gelatinase-associated lipocalin; *NGAL/Cr*, neutrophil gelatinase-associated lipocalin-to-creatinine ratio; *PP*, pulse pressure; *PREDICT*, Prospective study on the Early Detection and Identification of Cardiovascular disease and Hypertension; *SBP*, systolic blood pressure; *SD*, standard deviation; *SPRINT*, Systolic Blood Pressure Intervention Trial; *TRIBE-AKI*, Translational Research Investigating Biomarker End Points in Acute Kidney Injury; *uACR*, urinary albumin-to-creatinine ratio; *UMOD*, uromodulin; *UMOD/Cr*, uromodulin-to-creatinine ratio; *WIHS*, Women’s Interagency HIV Study

**Table 2:** Main characteristics of studies reporting associations between kidney biomarkers and markers of target organ damage

Study; Year (ref)	Country	Study design	Study population	Biomarker(s)	Population characteristics	Main findings	Notes
Ishiwata et al*.* 2021 [39]	Country, study design, study population, biomarkers and population characteristics are reported in Table 1					• Ascending quartiles of A1M/Cr: individuals in the highest A1M group were more likely to have higher LVEF (*p* = 0.002)	• Authors did not explore associations between LVEF and A1M in multiple regression analyses
Jiang et al. 2023 [54]	North America	Multicenter, prospective cohort study (follow-up 1 year)	CKiD participants	A1M indexed to urinary creatinine	Total study population^**#**^: • *N* = 504 • Age (years) = 12 (8–15) • Male (%) = 62 • Hypertension (%) = 17 • LVH prevalence (%, year after enrollment) = 12 • LVMI z-score = 0.15 (−0.74–1.04) • eGFR (ml/min/1.73m^2^) = 54 (41–68) • Urinary protein-to-creatinine ratio (g/g) = 0.32 (0.11–0.94)	• After multivariable adjustment for covariates, two-fold higher levels of A1M were associated with a lower prevalence of LVH (prevalence ratio: 0.90; 95% CI, 0.82 to 0.99; *p* = 0.026) • No associations were present between A1M and LVMI z-score	• Authors did not report median values of A1M • Authors assessed the cross-sectional association between the log_2_ biomarker levels and LVH using a Poisson regression model • Covariates included age, sex, race, body mass index, hypertension, urinary protein-to-creatinine ratio, glomerular diagnosis, and eGFR at study entry • LVH was defined as LVMI ≥ 95th percentile for healthy children
Wettersten et al. 2022 [55]	North America	Cross-sectional	MESA Participants without diabetes, CVD, or CKD	A1M and UMOD indexed to urinary creatinine	Total study population*: • *N* = 393 • Age (years) = 60 ± 10 • Male (%) = 48 • SBP (mmHg) = 122 ± 21 • DBP (mmHg) = 71 ± 11 • Hypertension (%) = 34 • Left ventricular mass-to-volume ratio (g/ml) = 0.93 ± 0.18 • LVEF (%) = 62 ± 6 • eGFR (ml/min/1.73m^2^) = 96 ± 16 • uACR (ug/mg)^**#**^ = 4.83 (3.10–9.20) • A1M/Cr (mg/mg)^**#**^ = 0.08 (0.05–0.12) • UMOD/Cr (pg/ml)^**#**^ = 262 429 (138 486–442 285)	• UMOD correlated negatively with Left ventricular mass-to-volume ratio (*r* = −0.13; *p* < 0.01) • In multivariable linear regression: no significant association between UMOD and Left ventricular mass-to-volume ratio or LVEF • No correlation or association between A1M and Left ventricular mass-to-volume ratio • Each twofold greater concentration of A1M was associated with a 1.2% lower LVEF (95% CI, −2.20 to −0.20; *p* < 0.05) • Individuals in the highest tertile of A1M had lower LVEF (*p* < 0.05)	• A1M and UMOD were entered into the multiple regression models without indexing for urine creatinine, but 1/urine creatinine was adjusted for in the models
Stopic et al. 2021 [56]	Serbia	Prospective cohort study (follow-up: 18 months)	Individuals over 18 years of age with CKD and an eGFR < 60 ml/min/1.73 m^2^	NGAL	Subgroup with CV events: • *N* = 25 • Age (years) = 68.1 ± 11.1 • Male (%) = 56 • SBP (mmHg) = 154 ± 8 • DBP (mmHg) = 93 ± 4 • Arterial hypertension (%) = 50 • NGAL (ng/ml) = 1.5 ± 0.5 Subgroup without CV events: • *N* = 26 • Age (years) = 65.4 ± 12.6 • Male (%) = 53.8 • SBP (mmHg) = 153 ± 5 • DBP (mmHg) = 94 ± 2 • Arterial hypertension (%) = 0 • NGAL (ng/ml) = 1.7 ± 0.5 Subgroup with LVH: • *N* = 22 • NGAL (ng/ml) = 1.6 ± 0.4 Subgroup without LVH: • *N* = 29 • NGAL (ng/ml) = 1.6 ± 0.6	• No significant difference was observed in NGAL regarding the presence/absence of CV events, or LVH	• To see the influence of newer biomarkers (including NGAL) on the occurrence of CV events, the authors did a comparative sub-analysis that included 51 patients who were at CKD stage 3b–5 hemodialysis • LVMI over 95 g/m^2^ for women and over 115 g/m^2^ for males were criteriums for LVH diagnosis • Authors did not explore associations between LVH and NGAL • Authors did not include urinary creatinine in any of the analyses

*Values expressed as mean ± standard deviation; ^**#**^ values expressed as median (Interquartile range), or frequency and percentage. Abbreviations: *A1M*, alpha-1 microglobulin; *A1M/Cr*, alpha-1 microglobulin-to-creatinine ratio; *CKD*, chronic kidney disease; *CKiD*, chronic kidney disease in children; *CV*, cardiovascular; *CVD*, cardiovascular disease; *DBP*, diastolic blood pressure; *eGFR*, estimated glomerular filtration rate; *LVEF*, left ventricular ejection fraction; *LVH*, left ventricular hypertrophy; *LVM*, left ventricular mass; *LVMI*, left ventricular mass index; *MESA*, multi-ethnic study of atherosclerosis; *NGAL*, neutrophil gelatinase-associated lipocalin; *SBP*, systolic blood pressure; *uACR*, urinary albumin-to-creatinine ratio; *UMOD*, uromodulin; *UMOD/Cr*, uromodulin-to-creatinine ratio

**Fig. 2:**
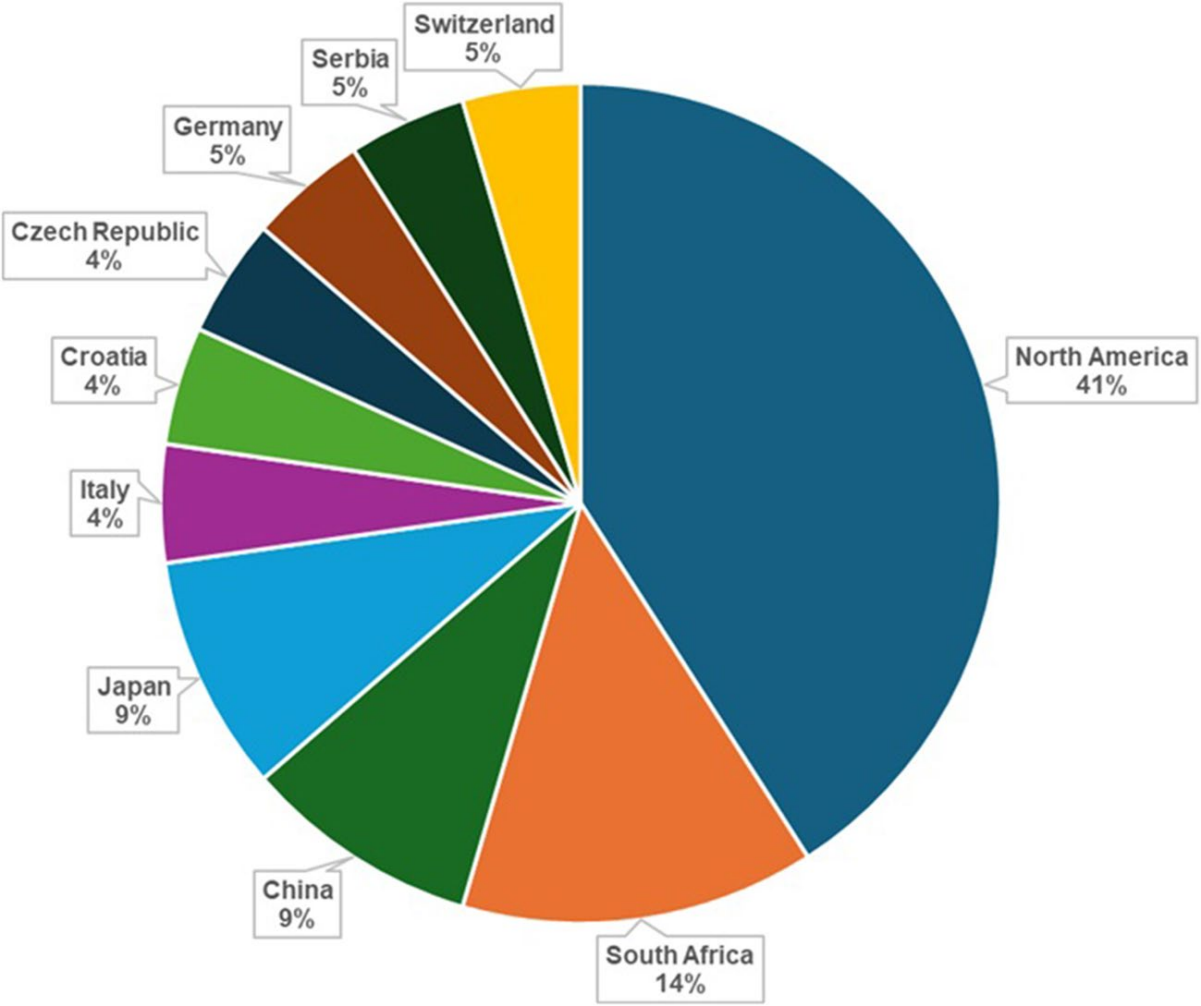
Percentage of studies done in different countries

**Fig. 3:**
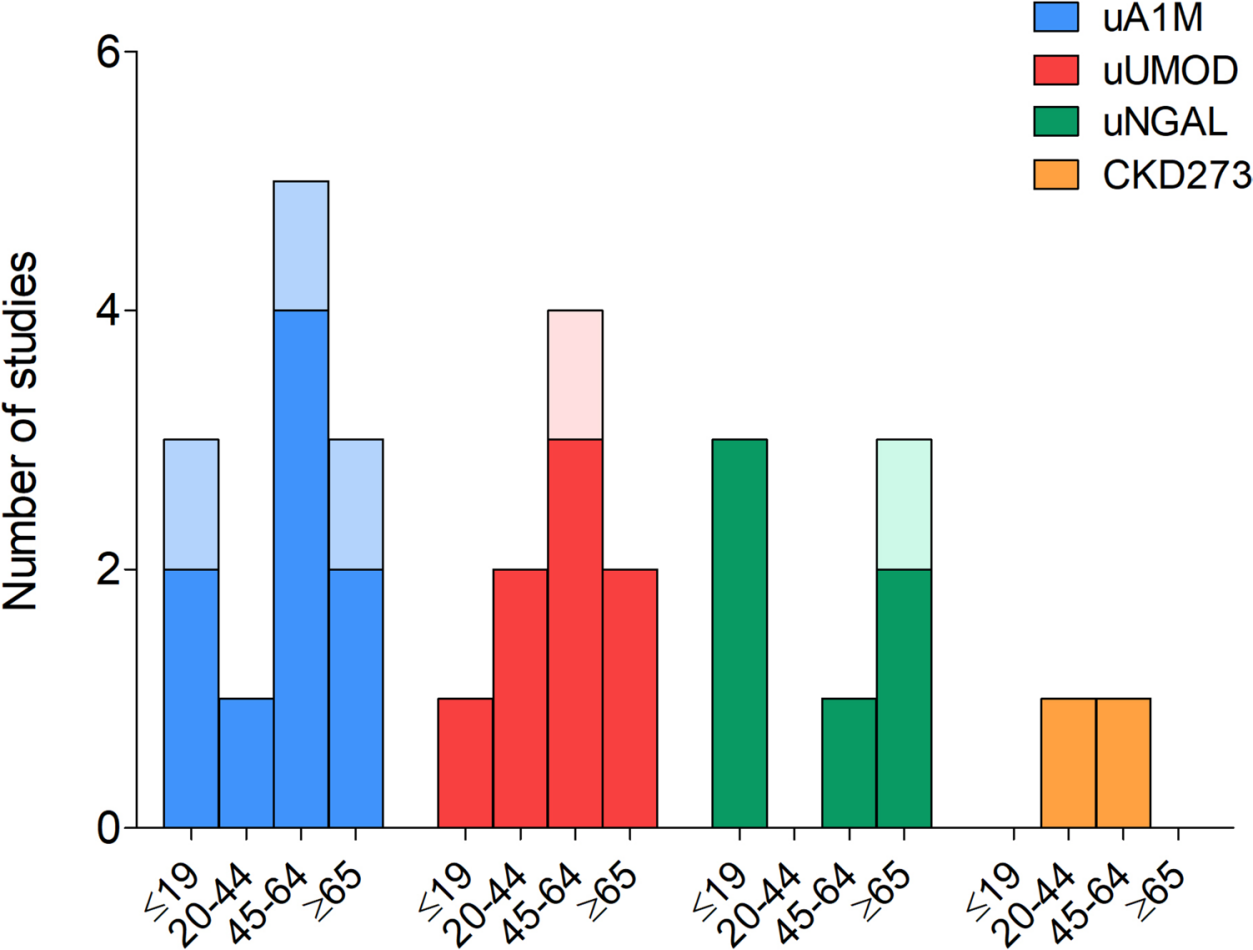
Number of studies in different age groups per kidney function biomarker. *A darker shade of colour indicates studies focusing on blood pressure, while a lighter shade of colour indicates studies focusing on markers of target organ damage.* Abbreviations: uA1M, urinary alpha-1 microglobulin; uNGAL, urinary neutrophil gelatinase-associated lipocalin; uUMOD, urinary uromodulin

The majority of studies identified had an observational design, including 12 cross-sectional [35, 36, 38, 42, 45–48, 50–52, 55], nine prospective [37, 39–41, 43, 49, 53, 54, 56] and one case control study [44]. Additionally, almost half of the studies (*n* = 9) were conducted in North America [36, 37, 43, 45, 48–50, 54, 55], followed by studies done in South Africa (*n* = 3) [35, 44, 47], China (*n* = 2) [40, 42], Japan (*n* = 2) [39, 51], while the remainder of the studies were conducted in Italy (*n* = 1) [41], Croatia (*n* = 1) [46], Czech Republic (*n* = 1) [38], Switzerland (*n* = 1) [52], Germany (*n* = 1) [53] and Serbia (*n* = 1) [56] (Fig. 2). Furthermore, the studies encompassed a wide range of age groups (Fig. 3), including seven studies in children and adolescents (aged ≤ 19 years) [35, 38, 43, 49, 50, 52, 54], two studies in young adults (aged between 20–44 years) [46, 47], seven studies in middle-aged adults (aged between 45–64 years) [37, 40, 41, 44, 48, 53, 55] and six studies in elderly individuals (aged ≥ 65 years) [36, 39, 42, 45, 51, 56].

The original and standardised quality scores of all the included studies are indicated in Supplementary Tables 2–4, Online Resource 1. The overall study quality was high, with a median quality score of 9 (range: 6–10). No low-quality studies were included.

### Associations Between Biomarkers of Kidney Function and Blood Pressure

Among the 19 studies that focused on associations with blood pressure, most included uA1M (*n* = 9) and uUMOD (*n* = 8) as biomarkers of kidney function, followed by uNGAL (*n* = 6), while CKD273 was the least investigated biomarker (*n* = 2).

*uA1M:* In an analysis performed in 957 children (Black; *n* = 526 and White; *n* = 431) from the Exercise, Arterial Modulation and Nutrition in Youth South Africa (ExAMIN Youth SA) study, Craig et al. [35] reported a positive association between urinary alpha-1 microglobulin-to-creatinine ratio (uA1M/Cr) and office systolic blood pressure (SBP; β = 0.15; 95% CI, 0.06 to 0.19; *p* < 0.001), diastolic blood pressure (DBP; β = 0.18; 95% CI, 0.09 to 0.24; *p* < 0.001) and mean arterial pressure (MAP; β = 0.11; 95% CI, 0.02 to 0.17; *p* = 0.018) in the Black group, as well as a positive association between uA1M/Cr and DBP (β = 0.10; 95% CI, 0.01 to 0.25; *p* = 0.038) in the White group. In the same study, it was found that the likelihood of elevated BP increased with 28% (Odds ratio = 1.28; 95% CI, 1.10 to 1.50) with each standard deviation increase in uA1M/Cr in the Black group (*p* = 0.002) [35]. In a large-scale study of 2436 participants in the Systolic Blood Pressure Intervention Trial (SPRINT) [36], it was found that each standard deviation increase in SBP at baseline (16 mmHg) was associated with higher levels of uA1M (10%) and lower levels of uUMOD (−2%) (all *p* < 0.05), while no association was observed with uNGAL. On the other hand, in a recent study by Khan et al. [37] with a median follow-up period of 9.9 years in 1170 adults in the Coronary Artery Risk Development in Young Adults (CARDIA) study, no statistically significant association of uA1M and uUMOD with incident hypertension or with a 10-year change in SBP or DBP was evident. Similarly, in a small-scale study on 37 children with Autosomal Dominant Polycystic Kidney Disease (ADPKD), Seeman et al*.* [38] found no significant correlation between uA1M/Cr and office blood pressure index.

In a study conducted by Ishiwata et al. [39] with a median follow-up period of 488 days and involving 623 adults with acute heart failure, it was observed that individuals in the higher quartiles of uA1M/Cr were more likely to have higher SBP (138 ± 27 mmHg vs 131 ± 27 mmHg; *p* = 0.013) and a history of hypertension (64.3% vs 44.4%; *p* = 0.002). In an analysis of 1451 participants enrolled in the Incidence, Development, and Prognosis of Diabetic Kidney Disease (INDEED) study with a 10-year follow-up, Sun et al. [40] observed a positive association between office SBP and uA1M levels (*p* < 0.01) and similarly, in logistic regression analyses, they found significant associations between the high stable SBP group and uA1M levels > 29.75 mg/L (Odds ratio = 2.91; 95%CI, 1.43 to 5.94; *p* < 0.05). Additionally, in a study with a 6-month follow-up (after the end of antiviral therapy), focusing on 135 Child–Pugh A cirrhotic patients receiving direct acting antiviral treatment for chronic hepatitis C, Bilioti et al. [41] indicated that the presence of hypertension with diabetes was associated with tubular dysfunction (Odds ratio = 4.23; 95%CI, 1.10 to 16.27; *p* < 0.05), indicated by uA1M/Cr levels > 14 µg/mg. Furthermore, in a study that involved 691 elderly community participants, Wang et al. [42] found that hypertension increased the likelihood of having elevated uA1M/Cr levels (> 15 mg/g) by 38% (Odds ratio = 1.38; 95% CI, 1.02 to 1.93; *p* = 0.011). These findings were further supported by the fact that 39.9% of individuals with hypertension and 47.2% of individuals with both hypertension and diabetes had increased uA1M/Cr levels in the same study [42].

*uUMOD:* In a study by Bakhoum et al. [43] with a median follow-up period of seven months that included 436 Chronic Kidney Disease in Children (CKiD) participants, univariate analyses showed that each two-fold higher urinary uromodulin-to-creatinine ratio (uUMOD/Cr) at baseline was associated with 1.66 mmHg (95% CI, −2.31 to −1.00) lower 24-h SBP (*p* < 0.001), 0.49 mmHg (95% CI, −0.97 to −0.01) lower 24-h DBP (*p* = 0.05), 1.71 mmHg (95% CI, −2.45 to −0.97) lower clinic SBP (*p* < 0.001) and 0.90 (95% CI, −1.59 to −0.22) lower clinic DBP (*p* = 0.01). However, these associations were attenuated with the inclusion of age in the multivariable models [43]. In a study population of 129 Black South Africans, including 71 individuals with clinically diagnosed hypertension-attributed chronic kidney disease (CKD), Nqebelele et al. [44] observed a negative correlation between uUMOD and office SBP (r = −0.23; *p* = 0.004). In this study, higher levels of uUMOD were also associated with 38% lower odds of having hypertension-attributed CKD (Odds ratio = 0.62; 95% CI, 0.48 to 0.81; *p* < 0.001) [44].

Additionally, in a study involving 933 participants from the Cardiovascular Health Study, Steubl et al. [45] found that the presence of hypertension was associated with lower uUMOD levels in univariate and multivariate (β = −4.30; 95% CI, −7.50 to −1.10; *p* < 0.05) analyses. In a recent study focusing on 326 young, middle-aged apparently healthy adults [46], uUMOD/Cr correlated negatively with several ambulatory blood pressure monitoring parameters in both prehypertensive and hypertensive groups. This included a negative correlation with 24-h and nighttime pulse pressure (PP) (Rho = −0.28; *p* = 0.04 and Rho = −0.31; *p* = 0.02, respectively) in the prehypertensive group as well as negative correlations with 24-h DBP (Rho = −0.29; *p* = 0.04), daytime DBP (Rho = −0.27; *p* = 0.04) and nighttime SBP (Rho = −0.29; *p* = 0.04) *p* = 0.03) in the hypertensive group [46]. While an analysis in 956 young apparently healthy adults from the African-Prospective study on the Early Detection and Identification of Cardiovascular Disease and Hypertension (African-PREDICT) study showed no significant associations of uUMOD/Cr and CKD273 classifier with office SBP [47]. In another study done by Muiru et al. [48] involving 198 human immunodeficiency virus (HIV)-positive individuals enrolled in the Multicenter AIDS Cohort Study (MACS) and the Women’s Interagency HIV Study (WIHS), no associations between uA1M, uNGAL or uUMOD and hypertension were observed.

*uNGAL:* In a study 5-year follow-up study involving 110 children with congenital heart disease enrolled in the Translational Research Investigating Biomarker End Points in AKI (TRIBE-AKI) study [49], no significant differences were found in 5-year median uNGAL concentrations or in the absolute change in uNGAL concentrations from baseline to five years in patients with hypertension compared to those without hypertension. However, in this study, hypertension was associated with a higher proportion of patients with 5-year uNGAL concentrations above normal for their specific age (10% vs 1%; *p* = 0.03) [49]. In addition, a study involving 31 participants, including 21 type-1 diabetes mellitus patients, Mamilly et al. [50] observed a negative correlation between urinary neutrophil gelatinase-associated lipocalin-to-creatinine ratio (uNGAL/Cr) and nocturnal SBP dipping (r = −0.47; 95% CI, −0.76 to −0.03; *p* < 0.05). On the other hand, a study that included 147 hypertensive patients, stratified according to eGFR values [51], found no significant correlation between uNGAL/Cr and BP. Similarly, in a small-scale study conducted on 30 children, including 15 children with ambulatory ADPKD [52], no association between uNGAL and MAP was present.

*CKD273:* In a small-scale study of 32 patients with resistant hypertension undergoing baroreflex activation therapy for six months, Wallbach et al. [53] found no correlation between changes in 24-h SBP values and changes in the urinary CKD273 score. In this study, the data was also stratified according to ambulatory blood pressure response and subsequently, there was a statistically significant (*p* = 0.011) reduction in the CKD273 score from a mean of 0.16 (95% CI, −0.16 to 0.56) to − 0.44 (95% CI, −0.63 to 0.05) after baroreflex activation therapy in patients with an ambulatory SBP decrease of ≥ 5 mm Hg [53].

### Associations Between Biomarkers of Kidney Function and Target Organ Damage

Among the four studies that focused on associations with markers of target organ damage, most studies included uA1M (*n* = 3), while the other studies focused on uUMOD (*n* = 1) and uNGAL (*n* = 1). No studies investigated CKD273 in this regard.

*uA1M*: Apart from observing an association between uA1M/Cr and blood pressure, Ishiwata et al. [39] also indicated that, when individuals with acute heart failure are stratified according to quartiles of uA1M/Cr, individuals in the higher uA1M/Cr group were more likely to have higher LVEF (55.5% vs 47%; *p* = 0.002). Another study involving 504 children enrolled in the CKiD study with a 1-year follow-up [54] found that a two-fold increase in uA1M levels was associated with a lower prevalence of left ventricular hypertrophy (LVH) (Prevalence ratio = 0.90; 95% CI, 0.82 to 0.99; *p* = 0.026). In 393 participants of the Multi-Ethnic Study of Atherosclerosis (MESA) without diabetes, CVD or CKD [55], each two-fold greater concentration of uA1M/Cr was associated with a 1.2% (95% CI, −2.20 to −0.20) lower LVEF (*p* < 0.05). The latter is further supported by the fact that, when stratified according to uA1M/Cr levels, individuals in the highest tertile of uA1M/Cr had lower LVEF (*p* < 0.05) [55].

*uUMOD*: In the MESA study [55], uUMOD/Cr also correlated negatively with left ventricular mass to volume ratio (r = −0.13; *p* < 0.01). However, these results were no longer significant in multivariable linear regression analyses after adjusting for age, gender, race, education site, blood pressure medications, SBP total cholesterol, lipid-lowering medication, body mass index, eGFR, urine albumin and 1/urine creatinine.

*uNGAL*: In a small-scale study by Stopic et al. [56] with a follow-up of 18 months in 51 individuals over 18 years of age with CKD, no significant differences were observed in uNGAL levels regarding the presence or absence of cardiovascular events or LVH.

**Fig. 4:**
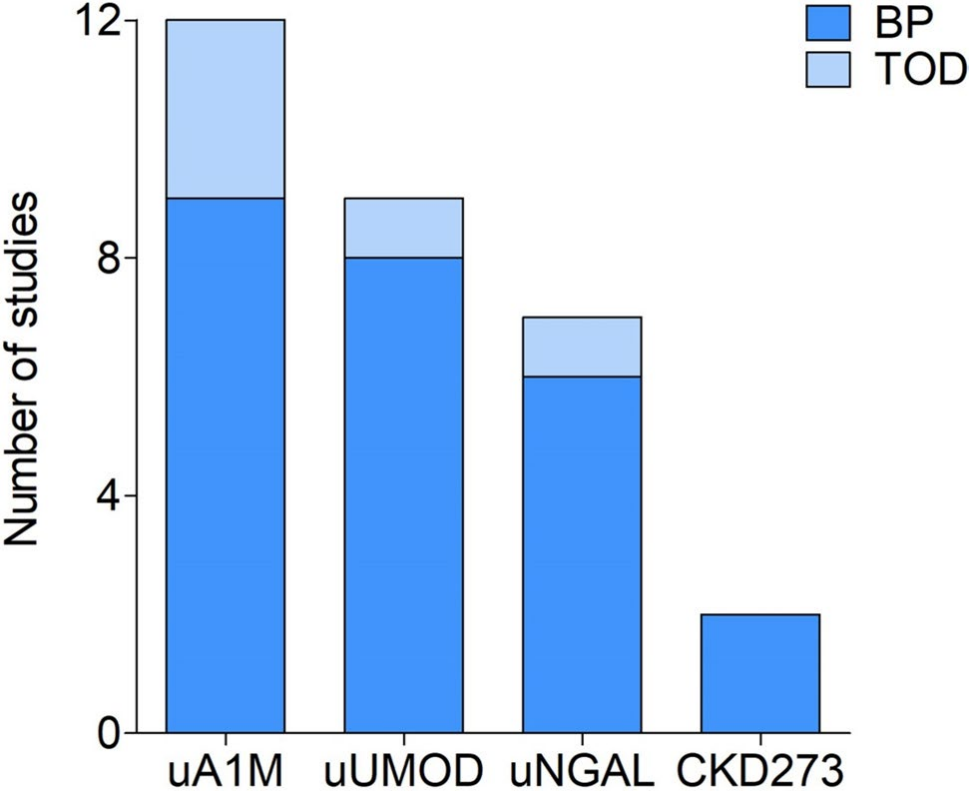
Number of studies that investigated each kidney function biomarker in relation to cardiovascular parameters. *A darker shade of colour indicates studies focusing on blood pressure, while a lighter shade of colour indicates studies focusing on markers of target organ damage.* Abbreviations: uA1M, urinary alpha-1 microglobulin; BP, blood pressure; uNGAL, urinary neutrophil gelatinase-associated lipocalin; TOD, target organ damage; uUMOD, urinary uromodulin

**Fig. 5:**
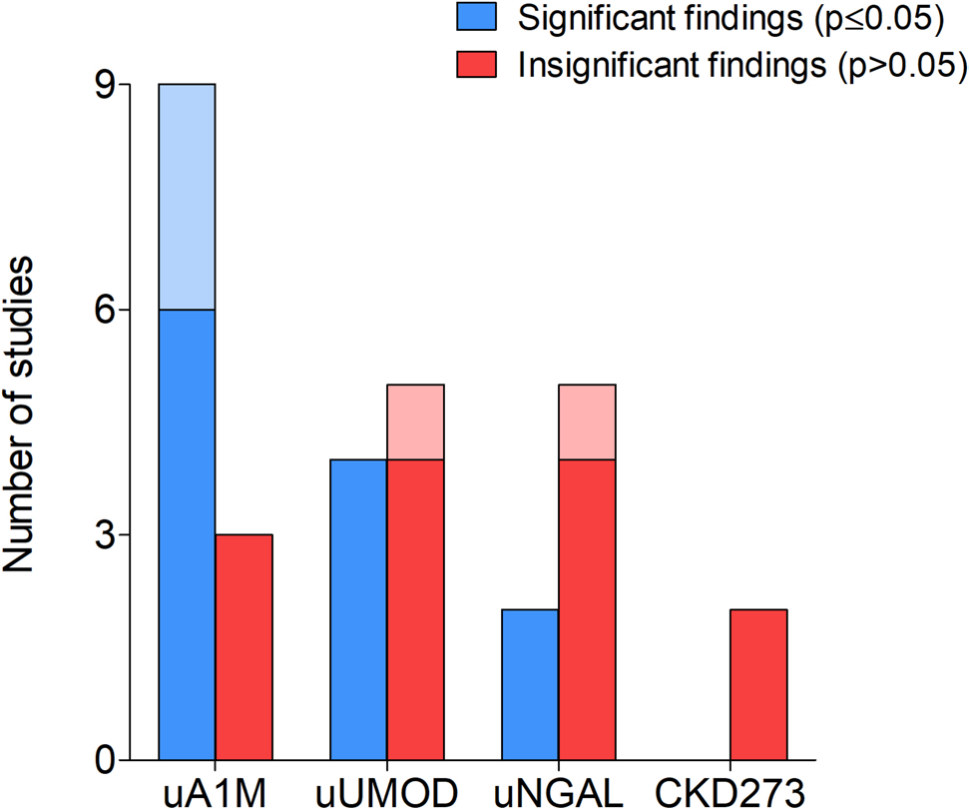
Number of studies with significant findings vs insignificant findings for each kidney function biomarker. *A darker shade of colour indicates studies focusing on blood pressure, while a lighter shade of colour indicates studies focusing on markers of target organ damage. Significant findings were defined as any correlations or associations between kidney function biomarkers and blood pressure or target organ damage markers with a p* ≤ *0.05.* Abbreviations: uA1M, urinary alpha-1 microglobulin; uNGAL, urinary neutrophil gelatinase-associated lipocalin; uUMOD, urinary uromodulin

Taking the above into account, it is evident that urinary kidney function biomarkers such as uA1M and uUMOD were more frequently studied in relation to cardiovascular health than uNGAL and CKD273 (Fig. 4). Furthermore, several conflicting results were observed in terms of significant and insignificant findings when exploring the correlations and associations between kidney function biomarkers and different measures of cardiovascular health (Fig. 5). The latter may be due to the various geographical regions, study designs, study populations, and age ranges that were investigated as well as whether the urinary biomarkers were adjusted for urinary creatinine concentration to account for urine tonicity.

## Discussion

Given that the kidneys play an important role in regulating blood pressure and maintaining cardiovascular health, investigating kidney-related biomarkers may aid in the identification of individuals who are at an increased risk of early CVD development and progression. This is particularly important due to the silent nature of hypertension and CVD development at an early and preventable stage when intervention will be the most effective. Therefore, in this systematic review, we aimed to explore associations between different urinary biomarkers of kidney function (uA1M, uNGAL, uUMOD and CKD273 classifier) and various cardiovascular measures (blood pressure and markers of target organ damage). Our review suggests that uA1M and uUMOD are more frequently studied and related to cardiovascular health than uNGAL or CKD273 classifier. Furthermore, our review highlights that, although a wide range of countries, study populations, ethnicities and age groups have been investigated, only a limited number of studies have focused on young and healthy South African adults. This is particularly concerning, given that this is a vulnerable population with an alarming prevalence of hypertension and CVD already evident in youth.

In six studies, ranging from children with CKD to elderly community individuals, uA1M was positively associated with SBP, DBP, MAP as well as the presence of hypertension [35, 36, 39–42]. Interestingly, all these findings were in terms of office blood pressure and none of the studies included 24-h ambulatory blood pressure measures. Furthermore, in four studies, uUMOD was inversely linked to office SBP, ambulatory blood pressure measures (24-h, daytime and nighttime) and hypertension [36, 44–46]. The majority of these studies included middle-aged adults (aged between 45–64 years) [44] and elderly individuals (aged ≥ 65 years) [36, 45]. In another three studies, uA1M was associated with markers of target organ damage – including LVEF and LVH [39, 54, 55]. In terms of LVEF, conflicting results were observed, since Ishiwata et al. [39] reported that individuals with higher uA1M had higher LVEF, while Wettersten et al. [55] indicated higher uA1M was associated with lower LVEF. In addition, Jiang et al. [54] found that increased uA1M was associated with a lower prevalence of LVH in children with CKD. The authors suggested that this unexpected association may represent a mechanistic pathway involving uA1M that is not yet understood, could reflect a chance finding or could be due to the young age group that was investigated [54].

Both uA1M and uUMOD are present at distinct parts of the nephron and have been proposed to provide novel insights into kidney tubule health independent of eGFR and uACR [23, 29, 57, 58]. Increased uA1M levels are an indication of proximal tubular dysfunction [25], while decreased uUMOD levels represent impaired tubular reserve and protein synthesis [59]. Since kidney tubule cells are responsible for maintaining water and electrolyte balance, mineral metabolism, acid–base regulation and ultimately urinary homeostasis, any disruptions in tubule function may have deleterious effects [29, 57, 60]. The latter is particularly relevant in the setting of cardiovascular health, as impaired sodium and water excretion can lead to volume overload, changes in neurohormonal balance, increased blood pressure and ultimately abnormalities in cardiac structure and function [28, 29, 36, 57]. Future studies are needed to investigate tubular dysfunction, represented by uA1M and uUMOD, as one of the possible predominant mechanisms linking early deterioration in kidney function to adverse alterations in cardiovascular health.

Furthermore, some studies observed no statistically significant associations of uA1M [37, 38, 48] and uUMOD [37, 43, 47, 48, 55] with blood pressure or markers of target organ damage. However, several of these studies were conducted in North America and the lack of associations may partly be explained by the fact that these participants were American rather than African individuals and therefore, the aetiology for essential hypertension may vary in different regions. A few studies only explored associations in small study populations with diseases such as ADPKD and HIV. In other studies, significant associations were attenuated when adjusting for potential confounders (traditional CVD risk factors, eGFR and albuminuria) in multivariable models. Collectively, these findings indicate there may still be several controversies about the role of uA1M and uUMOD in cardiovascular health and that specific study designs, populations and settings are needed to clarify this relationship.

Our analyses also identified other biomarkers and settings that warrant further investigation. Limited studies focused on urinary biomarkers of kidney function and markers of target organ damage. Although some significant associations were found in terms of uA1M, the results were conflicting and unexpected and should be validated in other populations and settings. Furthermore, most of these studies only explored associations in middle-aged adults and elderly individuals. It is noteworthy to mention that only measures of cardiac structure and function such as LVEF, LVM and LVH were explored and no studies included retinal microvascular parameters, arterial stiffness, or atherosclerosis. While uNGAL and the CKD273 classifier have been proposed as early biomarkers of deterioration in kidney function [26, 27], they were relatively understudied and not found to be related to cardiovascular health in our review. This may be partly due to the relatively small number of included studies, small sample sizes, different methods used to measure biomarkers and the fact that uNGAL and CKD273 can be regarded as biomarkers of kidney injury instead of kidney dysfunction. Additionally, the high cost of specialised equipment needed to analyse the CKD273 classifier may also be a reason why this sophisticated biomarker was understudied. Future studies should evaluate the clinical and prognostic performance of uNGAL and CKD273 with regard to cardiovascular health in alternative clinical contexts.

Our results should be viewed within the context of the strengths and limitations. We conducted a broad search in three large databases to capture the maximum number of applicable studies. Our inclusion criteria and evaluation of the literature were such that only high-quality studies were included in this systematic review. Our study provides a comprehensive systematic review of the literature regarding urinary biomarkers of kidney function and various measures of cardiovascular health. Despite broad search algorithms in PubMed, EBSCOhost and Scopus, the inherent time restriction of conducting a systematic review excluded any new studies published after December 2023. The included studies were also restricted to English and therefore, studies in other languages were not included. While discrepancies in the selection process were effectively resolved through comprehensive discussions between the two independent reviewers, a limitation of this review is the lack of formal inter-rater reliability metrics to quantify agreement between reviewers. In addition, although studies included in this systematic review were generally of high methodological quality, some biomarkers were only investigated in one or two studies or had small participant numbers. The latter has potential implications for the power, statistical significance and generalisability of the results. Future studies are needed to validate these findings in larger cohorts. In a few studies, there were differences in the measurements of urinary biomarkers and whether the biomarkers were indexed to urinary creatinine or adjusted for it in statistical analysis. Since urine tonicity and urinary flow can have an impact on biomarker collection, this could have influenced biomarker concentrations and the comparability of results across studies. Given the broad scope of kidney function biomarkers and cardiovascular health measures we included in our review, there was significant heterogeneity across the studies determined by study characteristics including study population size, age, race/ethnicity, male/female, pre-existing comorbidities, diseased conditions and biomarker transformation (for example, log values, quartiles, per standard deviation increase). Due to the observed heterogeneity across studies and absence of meta-analysis, we did not conduct a formal assessment of publication bias. While our findings included both significant and insignificant results, potentially reducing the risk of publication bias, this limitation should still be considered when interpreting our review’s conclusions. Although we reported and analysed each individual study’s results independently, examining multiple biomarkers and outcomes still increases the risk of false positive findings. Therefore, our results should be interpreted with caution. Furthermore, some studies only used unadjusted analyses when exploring the link between kidney function biomarkers and the different measures of cardiovascular health. Since there are various factors (traditional and non-traditional) that could influence cardiovascular health, this may result in biased estimates of associations, potentially limiting the validity of the results. Lastly, the variation in follow-up periods across individual cohorts could compromise the detection of cardiovascular health outcomes and impact the significance of the findings.

## Conclusion

Among the kidney function biomarkers included in this review, uA1M, followed by uUMOD, were studied more frequently and related to cardiovascular health. Apart from currently recognised clinical measures of kidney function (eGFR and uACR), urinary biomarkers such as uA1M and uUMOD may show promise as early indicators of kidney deterioration that should be considered in routine kidney and hypertension screening procedures.

## Supplementary Information

Below is the link to the electronic supplementary material.

Supplementary file1 (DOCX 30 KB)

## Data Availability

The datasets used and/or analysed during the current study are available from the corresponding author on reasonable request.
