# Supplementary material for: Urinary Biomarkers Of Kidney Function As Predictors Of Cardiovascular Health: A Systematic Review
Source: Curr Hypertens Rep. 2025 Feb 21;27(1):11. doi: 10.1007/s11906-025-01328-5 (PMC11845436; doi:10.1007/s11906-025-01328-5)
Supplement: Supplementary file 1 — Supplementary file1 (DOCX 30 KB) [file 11906_2025_1328_MOESM1_ESM.docx]

**Urinary Biomarkers Of Kidney Function As Predictors Of Cardiovascular Health: A Systematic Review**

A Degenaar^1^, R Kruger^1,2^, A Jacobs^1,2^, CMC Mels^1,2,^*

*^1^ Hypertension in Africa Research Team (HART), North-West University, Potchefstroom, South Africa*

*^2^ MRC Research Unit: Hypertension and Cardiovascular Disease, North-West University, Potchefstroom, South Africa*

***Corresponding author:**

Prof Catharina MC Mels Tel: +27 18 299 1983.

Hypertension in Africa Research Team (HART) Email: [carina.mels@nwu.ac.za](mailto:carina.mels@nwu.ac.za)

Private Bag X1290 ORCID: 0000-0003-0138-3341

North-West University

Potchefstroom

South Africa 2520

**The authors report no conflicts of interest.**

**Supplementary Table 1:** Search strategies in PubMed, EBSCOhost and Scopus databases

| **Database** | **Search string** |
| --- | --- |
| PubMed  (Title or Abstract) | ((Alpha 1 microglobulin[Title/Abstract]) OR (A1M[Title/Abstract]) OR (neutrophil gelatinase associated lipocalin[Title/Abstract]) OR (NGAL[Title/Abstract]) OR (lipocalin 2[Title/Abstract]) OR (UMOD[Title/Abstract]) OR (CKD273[Title/Abstract])) AND ((blood pressure[Title/Abstract]) OR (hypertension[Title/Abstract]) OR (elevated blood pressure[Title/Abstract]) OR (central retinal artery equivalent[Title/Abstract]) OR (CRAE[Title/Abstract]) OR (central retinal artery equivalent diameter[Title/Abstract]) OR (retinal arteriolar caliber[Title/Abstract]) OR (Central retinal vein equivalent[Title/Abstract]) OR (CRVE[Title/Abstract]) OR (central retinal vein equivalent diameter[Title/Abstract]) OR (retinal venular caliber[Title/Abstract]) OR (arteriolar-to-venular ratio[Title/Abstract]) OR (AVR[Title/Abstract]) OR (arteriole-to-venule ratio[Title/Abstract]) OR (retinal arteriovenous ratio[Title/Abstract]) OR (arteriovenous ratio[Title/Abstract]) OR (light flicker provocation[Title/Abstract]) OR (flicker light provocation[Title/Abstract]) OR (DVA[Title/Abstract]) OR (flicker-light-induced-provocation[Title/Abstract]) OR (FLIP[Title/Abstract]) OR (Retinal Vasculature Reactivity[Title/Abstract]) OR (retinal imaging[Title/Abstract]) OR (retinal vascular imaging[Title/Abstract]) OR (retinal microvasculature[Title/Abstract]) OR (retinal vasculature[Title/Abstract]) OR (retinal vessel calibres[Title/Abstract]) OR (retinal vessel calibers[Title/Abstract]) OR (retinal vessel[Title/Abstract]) OR (Retinal vascular calibres[Title/Abstract]) OR (retinal vascular calibers[Title/Abstract]) OR (retinal vascular caliber[Title/Abstract]) OR (Retinal arteriolar and venular caliber[Title/Abstract]) OR (Retinal vascular caliber changes[Title/Abstract]) OR (pulse wave velocity[Title/Abstract]) OR (pulse wave analysis[Title/Abstract]) OR (PWV[Title/Abstract]) OR (arterial stiffness[Title/Abstract]) OR (carotid intima-media thickness[Title/Abstract]) OR (CIMT[Title/Abstract]) OR (intima media thickness[Title/Abstract]) OR (atherosclerosis[Title/Abstract]) OR (left ventricular mass[Title/Abstract]) OR (LVM[Title/Abstract]) OR (relative wall thickness[Title/Abstract]) OR (RWT[Title/Abstract]) OR (cardiac structure[Title/Abstract]) OR (cardiac function[Title/Abstract]) OR (cardiac structure and function[Title/Abstract]) OR (left ventricular hypertrophy[Title/Abstract]) OR (LVH[Title/Abstract]) OR (left ventricular ejection fraction[Title/Abstract]) OR (LVEF[Title/Abstract]) OR (EF[Title/Abstract]) OR (left ventricle ejection fraction[Title/Abstract]) OR (fractional shortening[Title/Abstract]) OR (FS[Title/Abstract]) OR (E/e'[Title/Abstract]) OR (E/e' ratio[Title/Abstract]) OR (Early mitral inflow velocity and mitral annular early diastolic velocity ratio[Title/Abstract]) OR (early diastolic mitral inflow velocity to early diastolic mitral annulus velocity[Title/Abstract]) OR (E/A ratio[Title/Abstract]) OR (peak velocities of early and late diastolic filling ratio[Title/Abstract]) OR (early to atrial filling velocity ratio[Title/Abstract]) OR (systolic function[Title/Abstract]) OR (systolic dysfunction[Title/Abstract]) OR (diastolic function[Title/Abstract]) OR (diastolic dysfunction[Title/Abstract]))  Filters: Humans, English |
| EBSCOhost (Abstract) | AB ( “Alpha 1 microglobulin” OR A1M OR “neutrophil gelatinase associated lipocalin” OR NGAL OR “lipocalin 2” OR “uromodulin” OR UMOD OR “CKD273” ) AND AB ( “blood pressure” OR “hypertension” OR “elevated blood pressure” OR “central retinal artery equivalent” OR CRAE OR “central retinal artery equivalent diameter” OR “retinal arteriolar caliber” OR “Central retinal vein equivalent” OR CRVE OR “central retinal vein equivalent diameter” OR “retinal venular caliber” OR “arteriolar-to-venular ratio” OR AVR OR “arteriole-to-venule ratio” OR “retinal arteriovenous ratio” OR “arteriovenous ratio” OR “light flicker provocation” OR “flicker light provocation” OR DVA OR “flicker-light-induced-provocation” OR FLIP OR “Retinal Vasculature Reactivity” OR “retinal imaging” OR “retinal vascular imaging” OR “retinal microvasculature” OR “retinal vasculature” OR “retinal vessel calibres” OR “retinal vessel calibers” OR “retinal vessel” OR “Retinal vascular calibres” OR “retinal vascular calibers” OR “retinal vascular caliber” OR “Retinal arteriolar and venular caliber” OR “Retinal vascular caliber changes” OR “pulse wave velocity” OR “pulse wave analysis” OR PWV OR “arterial stiffness” OR “carotid intima-media thickness” OR CIMT OR “intima media thickness” OR “atherosclerosis” OR “left ventricular mass” OR LVM OR “relative wall thickness” OR RWT OR “cardiac structure” OR “cardiac function” OR “cardiac structure and function” OR “left ventricular hypertrophy” OR LVH OR “left ventricular ejection fraction” OR LVEF OR EF OR “left ventricle ejection fraction” OR “fractional shORtening” OR FS OR “E/e'” OR “E/e' ratio” OR “Early mitral inflow velocity and mitral annular early diastolic velocity ratio” OR “early diastolic mitral inflow velocity to early diastolic mitral annulus velocity” OR “E/A ratio” OR “peak velocities of early and late diastolic filling ratio” OR “early to atrial filling velocity ratio” OR “systolic function” OR “systolic dysfunction” OR “diastolic function” OR “diastolic dysfunction” )  Filters: Full text, English |
| Scopus (Title, Abstract or Keywords) | ( TITLE-ABS-KEY ( "Alpha 1 microglobulin" OR "A1M" OR "neutrophil gelatinase associated lipocalin" OR "NGAL" OR "lipocalin 2" OR "uromodulin" OR "UMOD" OR "CKD273" ) AND TITLE-ABS-KEY ( "blood pressure" OR "hypertension" OR "elevated blood pressure" OR "central retinal artery equivalent" OR "CRAE" OR "central retinal artery equivalent diameter" OR "retinal arteriolar caliber" OR "Central retinal vein equivalent" OR "CRVE" OR "central retinal vein equivalent diameter" OR "retinal venular caliber" OR "arteriolar-to-venular ratio" OR "AVR" OR "arteriole-to-venule ratio" OR "retinal arteriovenous ratio" OR "arteriovenous ratio" OR "light flicker provocation" OR "flicker light provocation" OR "DVA" OR "flicker-light-induced-provocation" OR "FLIP" OR "Retinal Vasculature Reactivity" OR "retinal imaging" OR "retinal vascular imaging" OR "retinal microvasculature" OR "retinal vasculature" OR "retinal vessel calibres" OR "retinal vessel calibers" OR "retinal vessel" OR "Retinal vascular calibres" OR "retinal vascular calibers" OR "retinal vascular caliber" OR "Retinal arteriolar and venular caliber" OR "Retinal vascular caliber changes" OR "pulse wave velocity" OR "pulse wave analysis" OR "PWV" OR "arterial stiffness" OR "carotid intima-media thickness" OR "CIMT" OR "intima media thickness" OR "atherosclerosis" OR "left ventricular mass" OR "LVM" OR "relative wall thickness" OR "RWT" OR "cardiac structure" OR "cardiac function" OR "cardiac structure and function" OR "left ventricular hypertrophy" OR "LVH" OR "left ventricular ejection fraction" OR "LVEF" OR "EF" OR "left ventricle ejection fraction" OR "fractional shortening" OR "FS" OR "E/e'" OR "E/e' ratio" OR "Early mitral inflow velocity and mitral annular early diastolic velocity ratio" OR "early diastolic mitral inflow velocity to early diastolic mitral annulus velocity" OR "E/A ratio" OR "peak velocities of early and late diastolic filling ratio" OR "early to atrial filling velocity ratio" OR "systolic function" OR "systolic dysfunction" OR "diastolic function" OR "diastolic dysfunction" ) ) AND PUBYEAR > 2017 AND ( LIMIT-TO ( DOCTYPE , "ar" ) ) AND ( LIMIT-TO ( LANGUAGE , "English" ) ) |

**Supplementary Table 2:** JBL critical appraisal checklist for analytical cross-sectional studies

| **Studies** | **Criteria** | | | | | | | | |
| --- | --- | --- | --- | --- | --- | --- | --- | --- | --- |
|  | **Were the criteria for inclusion in the sample clearly defined** | **Were the study subjects and the setting described in detail?** | **Was the exposure measured in a valid and reliable way?** | **Were objective, standard criteria used for measurement of the condition?** | **Were confounding factors identified?** | **Were strategies to deal with confounding factors stated?** | **Were the outcomes measured in a valid and reliable way?** | **Was appropriate statistical analysis used?** | **Total scores** |
| Craig *et al*. [35] | Y | Y | Y | Y | Y | Y | Y | Y | 8 |
| Degenaar *et al*. [47] | Y | Y | Y | Y | Y | Y | Y | Y | 8 |
| Hosohata *et al*. [51] | Y | Y | Y | Y | Y | Y | U | N | 6 |
| Ikeme *et al*. [36] | Y | Y | Y | Y | Y | Y | U | Y | 7 |
| Josipovic *et al*. [46] | Y | Y | Y | Y | Y | Y | Y | N | 7 |
| Mamilly *et al*. [50] | Y | Y | Y | Y | Y | Y | Y | N | 7 |
| Muiru *et al*. [48] | Y | Y | Y | Y | Y | Y | Y | Y | 8 |
| Nqebele *et al*. [44] | Y | Y | Y | Y | Y | Y | Y | Y | 8 |
| Seeman *et al*. [38] | Y | Y | Y | Y | Y | Y | Y | N | 7 |
| Steubl *et al*. [45] | Y | Y | Y | Y | N | Y | N | Y | 6 |
| Tsingos *et al*. [52] | Y | Y | Y | Y | N | N | Y | N | 5 |
| Wang *et al*. [42] | Y | Y | Y | Y | Y | Y | U | Y | 7 |
| Wettersten *et al*. [55] | Y | Y | Y | Y | Y | Y | Y | Y | 8 |

Note: Higher scores indicate a higher quality study.

Abbreviations: N, no; U, unclear; Y, yes.

**Supplementary Table 3:** Newcastle-Ottowa quality assessment scale for cohort/longitudinal studies

| **Studies** | **Criteria** | | | | | | | | |
| --- | --- | --- | --- | --- | --- | --- | --- | --- | --- |
|  | **Representativeness of the exposed cohort** | **Selection of the non-exposed cohort** | **Ascertainment of exposure** | **Demonstration that outcome of interest was not present at start of study** | **Comparability of cohorts based on the design or analysis** | **Ascertainment of outcome** | **Was follow-up long enough for outcomes to occur** | **Adequacy of follow-up of cohorts** | **Total points** |
| Bakhoum *et al*. [43] | √ | √ | √ | √ | √√ | √ | √ | √ | 9 |
| Billiotti *et al*. [41] | √ | √ | √ | √ | √√ | - | √ | √ | 8 |
| Greenberg *et al*. [49] | √ | √ | √ | √ | √ | √ | √ | - | 7 |
| Khan *et al*. [37] | √ | √ | √ | √ | √√ | √ | √ | √ | 9 |
| Sun *et al*. [40] | √ | √ | √ | √ | √ | √ | √ | √ | 8 |
| Wallbach *et al*. [53] | - | √ | √ | √ | √ | √ | √ | √ | 7 |
| Ishiwata *et al*. [39] | √ | √ | √ | √ | √ | √ | √ | √ | 8 |
| Jiang *et al*. [54] | √ | √ | √ | √ | √√ | √ | √ | √ | 9 |
| Stopic *et al*. [56] | √ | √ | √ | √ | - | √ | √ | √ | 7 |

Note: More points indicate a higher quality study.

**Supplementary Table 4:** Standardised quality scores for cross-sectional and cohort/longitudinal studies

| **Study** | **Study design** | **Original Score** | **Total Score** | **Standardised Score** |
| --- | --- | --- | --- | --- |
| Craig *et al*. [35] | Cross-sectional | 8 | 8 | 10 |
| Degenaar *et al*. [47] | Cross-sectional | 8 | 8 | 10 |
| Hosohata *et al*. [51] | Cross-sectional | 6 | 8 | 8 |
| Ikeme *et al*. [36] | Cross-sectional | 7 | 8 | 9 |
| Josipovic *et al*. [46] | Cross-sectional | 7 | 8 | 9 |
| Mamilly *et al*. [50] | Cross-sectional | 7 | 8 | 9 |
| Muiru *et al*. [48] | Cross-sectional | 8 | 8 | 10 |
| Nqebele *et al*. [44] | Cross-sectional | 8 | 8 | 10 |
| Seeman *et al*. [38] | Cross-sectional | 7 | 8 | 9 |
| Steubl *et al*. [45] | Cross-sectional | 6 | 8 | 8 |
| Tsingos *et al*. [52] | Cross-sectional | 5 | 8 | 6 |
| Wang *et al*. [42] | Cross-sectional | 7 | 8 | 9 |
| Wettersten *et al*. [55] | Cross-sectional | 8 | 8 | 10 |
| Bakhoum *et al*. [43] | Cohort study | 9 | 9 | 10 |
| Billiotti *et al*. [41] | Cohort study | 8 | 9 | 9 |
| Greenberg *et al*. [49] | Cohort study | 7 | 9 | 8 |
| Khan *et al*. [37] | Cohort study | 9 | 9 | 10 |
| Sun *et al*. [40] | Cohort study | 8 | 9 | 9 |
| Wallbach *et al*. [53] | Cohort study | 7 | 9 | 8 |
| Ishiwata *et al*. [39] | Cohort study | 8 | 9 | 9 |
| Jiang *et al*. [54] | Cohort study | 9 | 9 | 10 |
| Stopic *et al*. [56] | Cohort study | 7 | 9 | 8 |
